# AMPA and GABAA receptor nanodomains assemble in the absence of synaptic neurotransmitter release

**DOI:** 10.3389/fnmol.2023.1232795

**Published:** 2023-08-03

**Authors:** Harrison J. Ramsay, Sara E. Gookin, Austin M. Ramsey, Dean J. Kareemo, Kevin C. Crosby, Dominik G. Stich, Samantha S. Olah, Hannah S. Actor-Engel, Katharine R. Smith, Matthew J. Kennedy

**Affiliations:** ^1^Anschutz Medical Campus, Department of Pharmacology, University of Colorado, Aurora, CO, United States; ^2^Anschutz Medical Campus, Advanced Light Microscopy Core, University of Colorado, Aurora, CO, United States

**Keywords:** Synapse, neurotransmitter release, postsynaptic neurotransmitter receptors, AMPA receptor, GABA receptor, excitatory, inhibitory, nanodomain

## Abstract

Postsynaptic neurotransmitter receptors and their associated scaffolding proteins assemble into discrete, nanometer-scale subsynaptic domains (SSDs) within the postsynaptic membrane at both excitatory and inhibitory synapses. Intriguingly, postsynaptic receptor SSDs are mirrored by closely apposed presynaptic active zones. These trans-synaptic molecular assemblies are thought to be important for efficient neurotransmission because they concentrate postsynaptic receptors near sites of presynaptic neurotransmitter release. While previous studies have characterized the role of synaptic activity in sculpting the number, size, and distribution of postsynaptic SSDs at established synapses, it remains unknown whether neurotransmitter signaling is required for their initial assembly during synapse development. Here, we evaluated synaptic nano-architecture under conditions where presynaptic neurotransmitter release was blocked prior to, and throughout synaptogenesis with tetanus neurotoxin (TeNT). In agreement with previous work, neurotransmitter release was not required for the formation of excitatory or inhibitory synapses. The overall size of the postsynaptic specialization at both excitatory and inhibitory synapses was reduced at chronically silenced synapses. However, both AMPARs and GABA_A_Rs still coalesced into SSDs, along with their respective scaffold proteins. Presynaptic active zone assemblies, defined by RIM1, were smaller and more numerous at silenced synapses, but maintained alignment with postsynaptic AMPAR SSDs. Thus, basic features of synaptic nano-architecture, including assembly of receptors and scaffolds into trans-synaptically aligned structures, are intrinsic properties that can be further regulated by subsequent activity-dependent mechanisms.

## Introduction

Super resolution light microscopy techniques have revealed previously unappreciated molecular topography at excitatory, and inhibitory synapses (Fukata et al., 2013; MacGillavry et al., 2013; Nair et al., 2013; Tang et al., 2016; Biederer et al., 2017; Lee et al., 2017; Sinnen et al., 2017; Chen et al., 2018; Haas et al., 2018; Crosby et al., 2019; Goncalves et al., 2020; Ramsey et al., 2021; Gookin et al., 2022; Hruska et al., 2022; Sun et al., 2022). For example, rather than being evenly distributed across the postsynaptic membrane, AMPA-type glutamate receptors (AMPARs), and their associated scaffold proteins concentrate into subsynaptic domains (SSDs) (Fukata et al., 2013; MacGillavry et al., 2013; Nair et al., 2013). Intriguingly, postsynaptic AMPAR SSDs are often localized immediately opposite pre-synaptic neurotransmitter release sites, forming a trans-synaptic molecular “nanocolumn,” thought to be critical for efficient synaptic transmission (Tang et al., 2016; Biederer et al., 2017). More recent studies show these general principles may also apply to inhibitory synapses, with GABA_A_Rs concentrated into SSDs tightly associated with the scaffold protein gephyrin, and in trans-synaptic alignment with putative presynaptic active zones (Pennacchietti et al., 2017; Crosby et al., 2019; Yang et al., 2021).

How does activity influence synaptic nano-architecture? Pioneering studies have demonstrated that synapse formation does not require neurotransmission (Verhage et al., 2000; Varoqueaux et al., 2002; Harms et al., 2005; Harms and Craig, 2005). Less is known, however, about the impact of synaptic activity on molecular nano-architecture prior to, and during the period of synapse development. Previous studies at mature excitatory and inhibitory synapses show that AMPARs, PSD95, and GABA_A_R/Gephyrin SSDs are altered in size, and/or number during various forms of plasticity, suggesting that activity-dependent remodeling of these assemblies is important for synaptic function (MacGillavry et al., 2013; Tang et al., 2016; Hruska et al., 2018; Crosby et al., 2019; Hruska et al., 2022; Sun et al., 2022). For example, ~48 h exposure to tetrodotoxin (TTX), which blocks evoked neurotransmitter release, results in expanded area of the postsynaptic density (PSD), and an increase in the number of PSD95 SSDs at individual synapses (MacGillavry et al., 2013). Similar experiments pharmacologically blocking AMPARs over longer time periods (~8 days) also found increases in synaptic PSD95 SSD area and number (Sun et al., 2022).

From these observations, it is clear that scaffold SSDs are maintained and can even form, independently of evoked neurotransmitter release or AMPAR activation. However, in previous work, manipulations were performed during or following the establishment of synaptic nano-architecture. Thus, it remains unclear whether neurotransmitter signaling is required at any point before or during the period of synapse formation for the development of postsynaptic neurotransmitter receptor SSDs, and/or their trans-synaptic alignment with presynaptic active zone structures.

Here, we utilized lentivirally-expressed tetanus neurotoxin (TeNT) (Ehlers et al., 2007; Lee et al., 2010) to permanently block evoked and spontaneous neurotransmitter release from pre-synaptic terminals of excitatory and inhibitory neurons prior to synaptogenesis. Despite the chronic lack of synaptic neurotransmitter release, AMPAR and GABA_A_R SSDs were observed at excitatory and inhibitory synapses, respectively. Although receptors and scaffolds assembled into SSDs in the absence of neurotransmitter release, total postsynaptic size was reduced at both excitatory and inhibitory synapses. At excitatory synapses, individual PSD95 SSDs were larger, but decreased in number, leading to a reduction in overall PSD95 area. For AMPARs, we observed no differences in the summed synaptic SSD area, individual SSD area, or number of SSDs per synapse. In TeNT-silenced presynaptic terminals, the active zone marker RIM1 formed SSDs that were smaller and more numerous than controls, but which remained in close apposition to postsynaptic AMPAR SSDs. At inhibitory synapses, the average size, but not number, of gephyrin SSDs was reduced, leading to smaller postsynaptic volume. Additionally, the summed GABA_A_R SSD volume per synapse was decreased. Collectively, these experiments reveal that basic features of synaptic nano-architecture, including coalescence into trans-synaptically aligned SSDs, are intrinsic properties that occur in the absence of neurotransmission, but can be sculpted by subsequent activity-dependent mechanisms.

## Results

### Validation of TeNT approach for disrupting neurotransmission prior to synapse development

**Figure 1:**
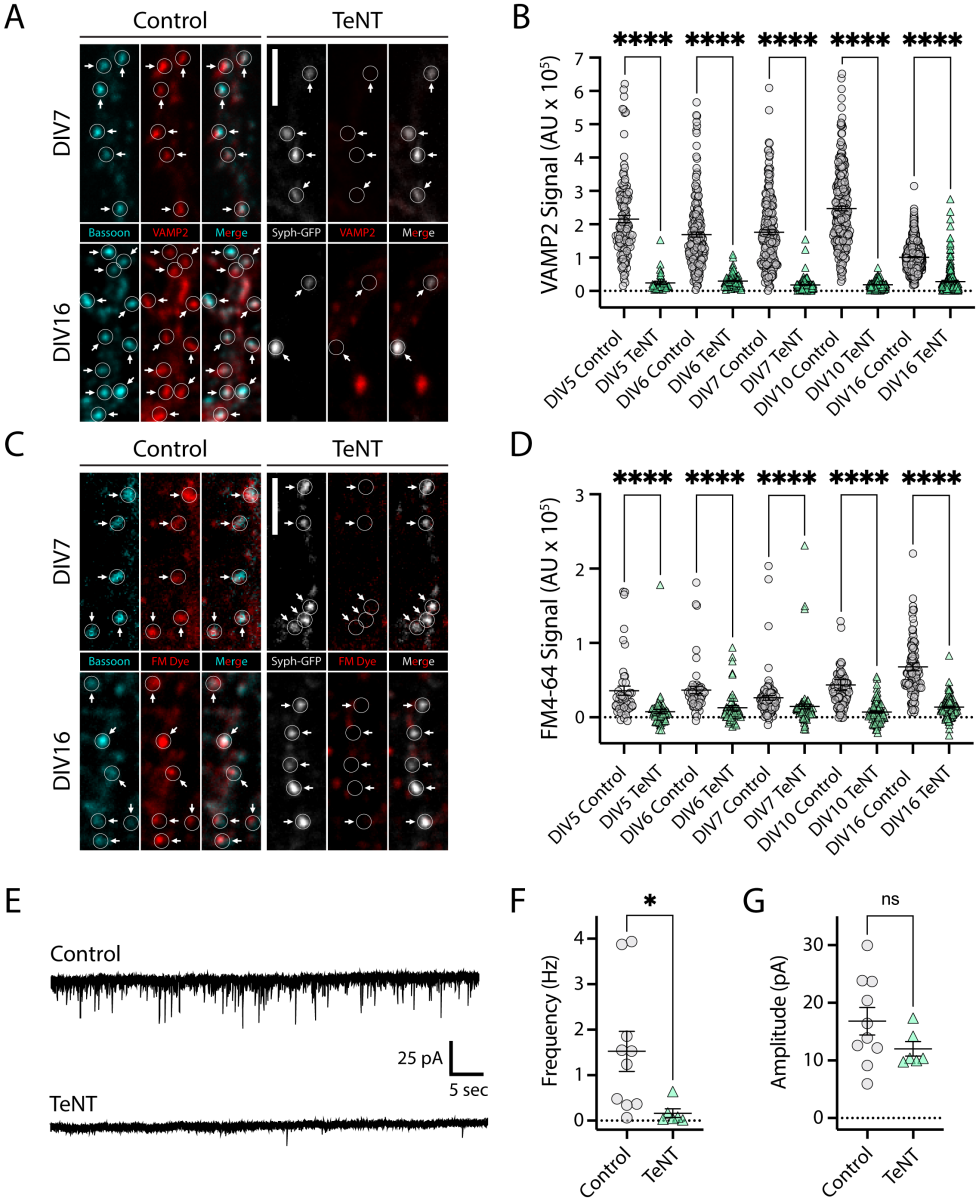
Validation of TeNT approach for disrupting neurotransmission prior to synapse development. **(A)** Images of dissociated hippocampal neurons immunostained with a VAMP2 antibody that does not recognize TeNT-cleaved VAMP2 (red). Control samples were co-stained for an independent presynaptic marker, bassoon (cyan). Note that VAMP2 staining is absent from syph-GFP-IRES-TeNT expressing presynaptic terminals at both DIV7 (arrows, top-right) and DIV16 (arrows, bottom-right) compared to uninfected control cultures (left, top/bottom). Bassoon (cyan), VAMP2 (red), syph-GFP-IRES-TeNT (greyscale). Scale bar = 5 μm. **(B)** Quantification of VAMP2 terminal fluorescence at syph-GFP-IRES-TeNT-expressing terminals at different timepoints throughout synaptic development. DIV5 (terminals, *n* = 126 control/35 TeNT), DIV6 (terminals, *n* = 197 control/84 TeNT), DIV7 (terminals, *n* = 209 control/138 TeNT), DIV10 (terminals, *n* = 270 control/179 TeNT), DIV16 (terminals, *n* = 848 control/322 TeNT); ****p < 0.0001 [Welch’s *T*-Test (DIV5, 6, 7, 10), Mann–Whitney (DIV16)]. **(C)** Syph-GFP-IRES-TeNT expressing presynaptic terminals (right, top/bottom) fail to load FM4-64 dye at both DIV7 (top-right), and DIV16 (bottom-right) timepoints, whereas uninfected control terminals (left, top/bottom) are robustly labeled. Bassoon (cyan), FM4-64 (red), syph-GFP-IRES-TeNT (greyscale). Scale bar = 5 μm. **(D)** Quantification of FM dye terminal fluorescence at syph-GFP-IRES-TeNT-expressing terminals at different timepoints throughout development. DIV5 (terminals, *n* = 48 control/66 TeNT), DIV6 (terminals, *n* = 53 control/53 TeNT), DIV7 (terminals, *n* = 89 control/89 TeNT), DIV10 (terminals, *n* = 99 control/99 TeNT), DIV16 (terminals, n = 107 control/107 TeNT); ****p < 0.0001 [Mann–Whitney (DIV5, 6, 7), Welch’s *T*-Test (DIV10, 16)]. **(E)** Example mEPSC recordings from control cultures (top), and cultures infected with syph-GFP-IRES-TeNT lentivirus (bottom). **(F)** Frequency of mEPSCs was significantly reduced in DIV9 cultures expressing syph-GFP-IRES-TeNT compared to controls [neurons, *n* = 10 control/6 TeNT; *p < 0.05 (*T*-Test)]. **(G)** Amplitude of remaining mEPSCs was unaffected in cultures infected with syph-GFP-IRES-TeNT lentivirus [neurons, *n* = 10 control/6 TeNT; ns, not significant (*T*-Test)].

To investigate synaptic nano-architecture in the absence of neurotransmission, we infected neurons with a lentiviral vector encoding TeNT, and synaptophysin-GFP (syph-GFP) separated by an internal ribosome entry site (IRES), as previously described (Ehlers et al., 2007; Lee et al., 2010). TeNT potently blocks neurotransmission through the proteolytic cleavage of vesicular soluble N-ethylmaleimide-sensitive factor attachment protein receptor (v-SNARE) proteins (Schiavo et al., 1992) critical for fusion of synaptic neurotransmitter vesicles at both excitatory, and inhibitory synapses. Co-expression of syph-GFP allows for fluorescence-based identification of terminals from TeNT-expressing neurons. To confirm that neurotransmission was blocked prior to synapse formation, it was important to first determine the time-course of TeNT expression and function at axonal terminals. We infected dissociated rat hippocampal neuronal cultures at day *in vitro* 1 (DIV1), with low viral titer to achieve sparse syph-GFP labeling of synaptic terminals. To assay TeNT activity, we fixed and stained the cultures for VAMP2, a major TeNT substrate, using an antibody that does not recognize TeNT-cleaved VAMP2. We quantified VAMP2 staining at several timepoints prior to (DIV5, 6), during (DIV7, 10) and following (DIV16) synaptogenesis. VAMP2 signal was nearly eliminated in TeNT-expressing terminals at all timepoints, compared to VAMP2 staining in age-matched, uninfected control terminals (Figures 1A,B). Thus, TeNT is expressed and active at synaptic terminals, even at timepoints prior to synapse formation. To confirm neurotransmitter vesicle fusion was blocked in terminals from syph-GFP-IRES-TeNT infected neurons, we performed styryl fluorescent dye (FM4-64) loading to stain functional terminals at the same timepoints as in our VAMP2 ICC experiments. Here we used a brief depolarizing stimulus (exposure to 50 mM KCl in iso-osmotic ACSF) to trigger neurotransmitter vesicle fusion, followed by a period of compensatory endocytosis, with activated terminals incorporating FM4-64 (Betz and Bewick, 1992). At each of our five timepoints, we observed drastically reduced FM4-64 fluorescence in TeNT-expressing terminals, confirming robust blockade of neurotransmission throughout the time period of synapse formation in our culture system (Figures 1C,D). Finally, as an independent functional measure, we infected a set of cultures with syph-GFP-IRES-TeNT virus on DIV1 and performed whole cell voltage clamp recordings of miniature excitatory post-synaptic currents (mEPSCs) at DIV7 and 9. Here we used a higher viral titer, to ensure silencing at most terminals. At DIV 7, we did not observe mEPSCs in uninfected control neurons, confirming that few, if any functional synapses had formed at this time point in our cultures. At DIV9, we observed reliable mEPSCs in control cultures, but mEPSCs were almost completely eliminated in syph-GFP-IRES-TeNT infected cultures (Figures. 1E,F). The average amplitude of the few remaining mEPSCs was not affected (Figure 1G). Together, these results demonstrate that our strategy effectively blocks neurotransmitter release prior to synapse development.

### Formation of excitatory receptor and scaffold SSDs in the absence of neurotransmission

**Figure 2:**
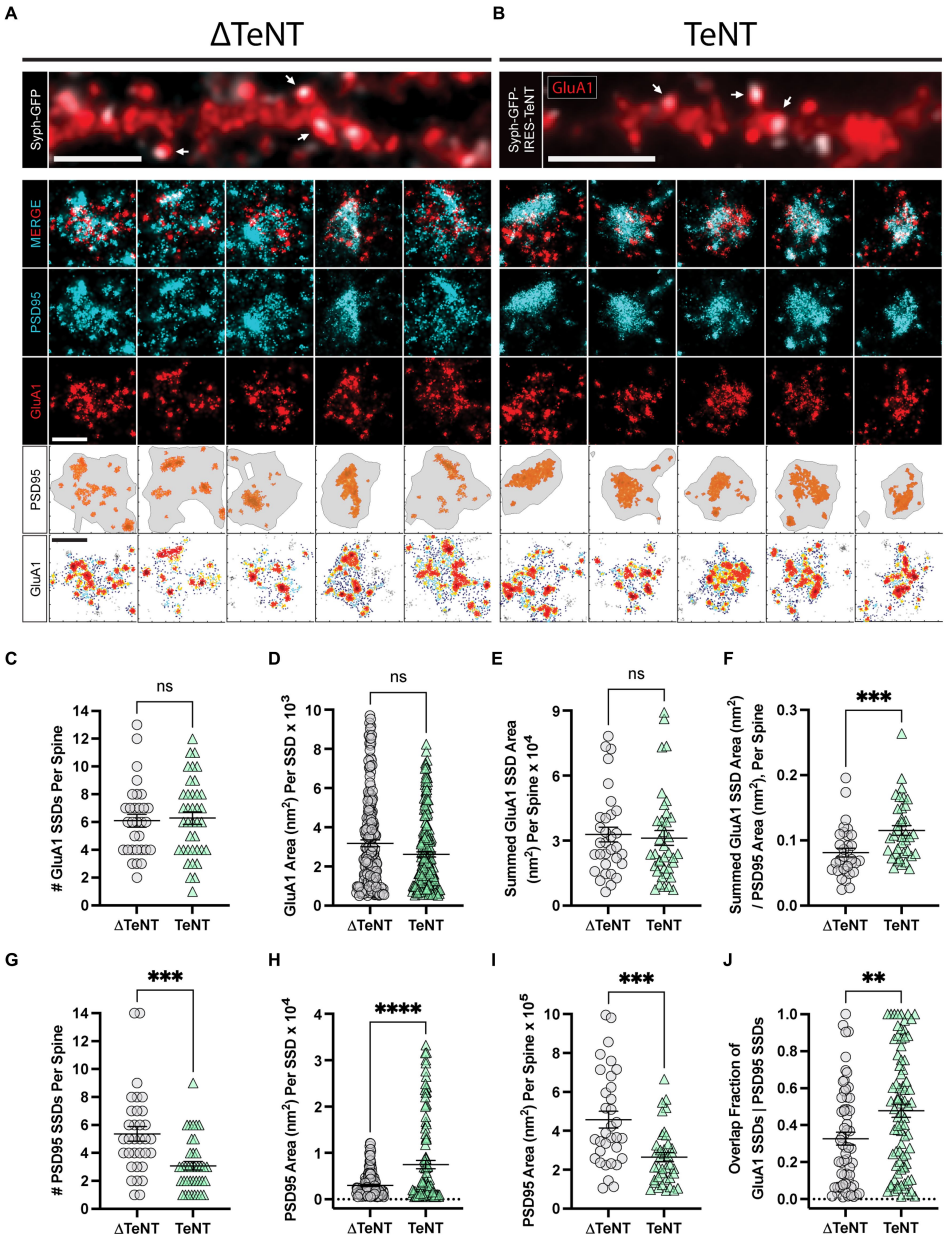
Formation of excitatory receptor and scaffold SSDs in the absence of neurotransmission. **(A,B)** Representative epifluorescence images of dendritic regions for cultures infected with control virus (∆TeNT, **A**), or TeNT virus (TeNT, **B**) and stained for surface GluA1 (red) are shown (top panels) with syph-GFP, or syph-GFP-IRES-TeNT shown in greyscale. In both cases note the apposition of syph-GFP signal and GluA1-positive (red) dendritic spines (white arrows). Scale bar = 5 μm in each image. Representative dSTORM images of synapses contacted by ∆TeNT **(A)**, or TeNT **(B)** terminals are shown below in rows 2–4. Row 5 shows the PSD boundary for each synapse, defined by a minimum PSD95 localization density (grey borders), with PSD95 localizations in defined SSDs rendered in orange. Row 6 shows GluA1 localization density maps for each synapse with warmer colors representing higher GluA1 localization density. Scale bars in rows 4, and 6 both = 500 nm. **(C)** There was no significant difference in the number of GluA1 SSDs per spine at TeNT-silenced synapses compared to controls [spines, *n* = 31 ∆TeNT/38 TeNT; ns, not significant; *p* = 0.7690 (*T*-Test)]. **(D)** The area of individual GluA1 SSDs was not significantly different at TeNT-silenced synapses compared to controls [SSDs, *n* = 194 ∆TeNT/200 TeNT; ns, not significant; *p* = 0.0531 (Mann–Whitney)]. **(E)** The summed GluA1 SSD area, per spine was not significantly different at TeNT-silenced synapses compared to controls [spines, *n* = 32 ∆TeNT/38 TeNT; ns = not significant; *p* = 0.5777 (Mann–Whitney)]. **(F)** The ratio between the summed GluA1 SSD area to its total PSD95 area was significantly increased at TeNT-contacted dendritic spines [spines, *n* = 33 ∆TeNT/37 TeNT; ***p < 0.001; *p* = 0.0007 (Mann–Whitney)]. **(G)** The number of PSD95 SSDs per spine was significantly decreased at TeNT-contacted spines compared to controls [spines, *n* = 33 ∆TeNT/38 TeNT; ***p < 0.001; *p* = 0.0002 (Mann–Whitney)]. **(H)** The area of individual PSD95 SSDs was significantly increased at TeNT-silenced spines compared to controls [SSDs, *n* = 141 ∆TeNT/103 TeNT; ****p < 0.0001 (Welch’s *T*-Test)]. **(I)** The total PSD95 area per spine was significantly reduced at TeNT-contacted spines compared to controls [spines, *n* = 33 ∆TeNT/38 TeNT; ***p = 0.0001 (Mann–Whitney)]. **(J)** The degree of GluA1 SSD/PSD95 SSD overlap was significantly increased at TeNT-contacted spines compared to controls [SSDs, *n* = 61 ∆TeNT/81 TeNT; **p < 0.01; *p* = 0.0052 (Mann–Whitney)].

We next tested whether AMPARs concentrate into SSDs at PSDs opposing silenced terminals. We used direct stochastic optical reconstruction microscopy (dSTORM) (Rust et al., 2006; Heilemann et al., 2008), to analyze postsynaptic proteins PSD95 and GluA1 at DIV16. One potential advantage of our TeNT-silencing approach is that we could in principle, directly compare silenced synapses to neighboring control synapses on the same dendrites. However, through validating this approach, we observed that many VAMP2-negative terminals contained undetectable syph-GFP signal (data not shown). This suggests that TeNT can actively cleave VAMP2 at terminals where the GFP reporter level is below the detection threshold. To circumvent this issue, comparisons were made between neuronal cultures infected with lentivirus that expressed either syph-GFP (viral control, ∆TeNT condition), or syph-GFP-IRES-TeNT (activity-silenced, TeNT condition). This allowed us to avoid including false-negative (i.e., TeNT positive with low or undetectable Syph-GFP) synapses in our dataset. This also controlled for any potential preference for lentiviral infection of specific neuronal subtypes that may generate synapses with distinct size, morphology or molecular nanoarchitecture. To visualize surface AMPARs, we incubated live neurons with an antibody against the AMPAR subunit GluA1 followed by fixation, permeabilization and labeling for the post-synaptic excitatory scaffolding protein PSD95. Dendritic spines adjacent to syph-GFP or syph-GFP-IRES-TeNT expressing terminals could be easily identified by epifluorescence imaging prior to dSTORM acquisitions (Figures 2A,B). Despite chronic blockade of neurotransmitter release, PSD95 and AMPAR SSDs clearly formed at TeNT-associated PSDs (Figure 2B). We found no differences in the number or area of individual GluA1 SSDs per synapse between control and TeNT-silenced conditions (Figures 2C,D). Accordingly, there was no significant difference in the summed GluA1 SSD area per spine (Figure 2E). While we observed no change in GluA1 nano-structure at silenced synapses, we did observe a significant increase in the area of individual PSD95 SSDs and a significant decrease in their number, per spine (Figures 2G,H). Even though individual SSDs were larger at TeNT-silenced synapses, their decreased number led to significantly smaller total PSD95 compartment areas (i.e., region encompassing all synaptic PSD95, defined by a minimum density of single molecule localizations) (Figure 2I). Given the smaller total PSD area at silenced synapses, a larger percentage of the PSD was occupied by GluA1 SSDs (Figure 2F). Finally given the more compact PSD, activity-silencing led to a significant increase in the overlap of individual GluA1 and PSD95 SSDs (Figure 2J). Together, these results demonstrate that neurotransmission is not required for the establishment of AMPAR or PSD95 SSDs, but plays a critical role in regulating the number, and size of scaffolding domains, as well as the overall size of the PSD.

### Trans-synaptic SSD alignment is intact at chronically silenced excitatory synapses

**Figure 3:**
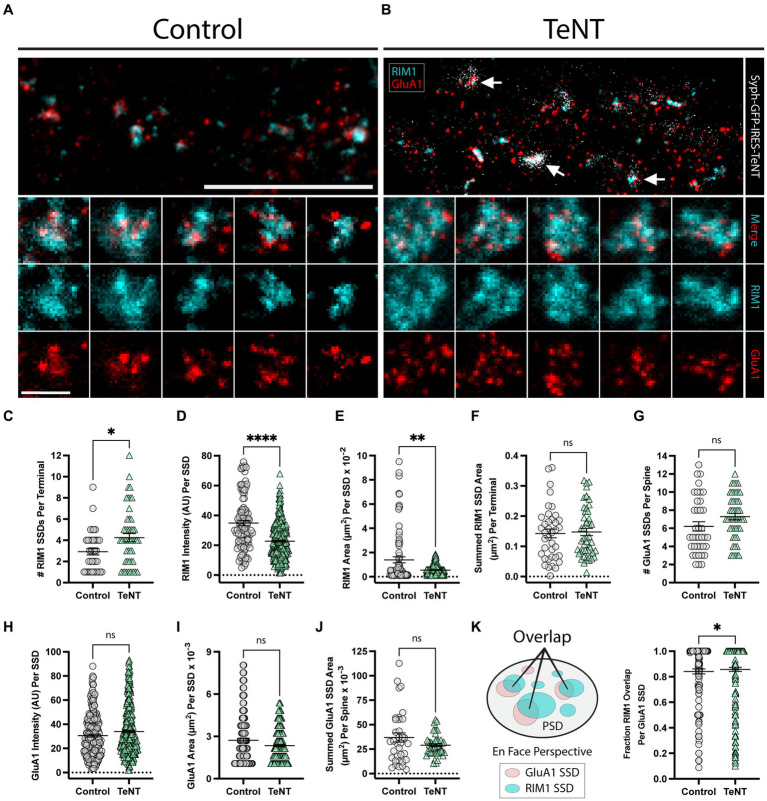
Trans-synaptic SSD alignment is intact at chronically silenced excitatory synapses. **(A,B)** Representative STED images of synapses labeled with RIM1 (cyan) and GluA1 (red) for (control, **A**) and (TeNT, **B**) conditions. Note the apposition of syph-GFP signal and GluA1-positive (red) dendritic spines (white arrows). Scale bar = 5 μm. Columns 1–5 in **(A,B)** display representative STED images of synapses for control **(A)**, and TeNT **(B)** conditions; merged RIM1 (cyan), and GluA1 (red) (row 2); RIM1 (cyan) (row 3); GluA1 (red) (row 4). Scale bar = 500 nm. **(C)** The number of RIM1 SSDs per synaptic terminal was significantly increased at TeNT-expressing terminals compared to controls [terminals, *n* = 38 control/49 TeNT; *p < 0.05 (Mann–Whitney)]. **(D)** RIM1 fluorescence intensity quantified at individual SSDs was decreased at TeNT-silenced terminals compared to controls [SSDs, *n* = 104 control/346 TeNT; ****p < 0.0001 (Mann–Whitney)]. **(E)** The area of individual RIM1 SSDs was decreased at TeNT-silenced terminals compared to controls [SSDs, *n* = 81 control/281 TeNT; **p < 0.01 (Welch’s *T*-Test)]. **(F)** The summed RIM1 SSD area per terminal was not significantly different at TeNT-silenced terminals compared to controls [terminals, *n* = 38 control/48 TeNT; ns, not significant; *p* = 0.7050 (Mann–Whitney)]. **(G)** The number of GluA1 SSDs per spine was not significantly different at TeNT-silenced spines compared to controls [spines, *n* = 38 control/49 TeNT; ns, not significant; *p* = 0.0539 (Mann–Whitney)]. **(H)** The fluorescence intensity of individual GluA1 SSDs was not significantly different at TeNT-silenced spines compared to controls [SSDs, *n* = 218 control/372 TeNT; ns, not significant; *p* = 0.0959 (Mann–Whitney)]. **(I)** The mean area of individual GluA1 SSDs was not significantly different at TeNT-silenced spines compared to controls [SSDs, *n* = 187 control/339 TeNT; ns, not significant; *p* = 0.2098 (Mann–Whitney)]. **(J)** The summed GluA1 SSD area per spine was not significantly different at TeNT-silenced spines compared to controls [spines, *n* = 35 control/49 TeNT; ns, not significant; *p* = 0.1120 (Welch’s *T*-Test)]. **(K)** Schematic showing the fraction of GluA1/RIM1 SSD overlap (left). GluA1 SSD overlap with RIM1 SSDs was slightly elevated at TeNT-associated synapses (i.e., on average, a larger area of each GluA1 SSD at TeNT-associated synapses was overlapped by closely-associated RIM1 SSDs) [SSDs, *n* = 155 Control/268 TeNT; *p < 0.05; *p* = 0.0372 (Mann–Whitney)].

We next measured whether chronic blockade of synaptic transmission impacted presynaptic active zone assembly and/or trans-synaptic AMPAR alignment. Many presynaptic proteins, most notably those that play key roles in vesicle docking and fusion in the presynaptic terminal, also concentrate into subsynaptic nanoscale structures (Tang et al., 2016). For example the presynaptic active zone protein RIM1 is observed in subsynaptic assemblies closely aligned with PSD95, and AMPAR SSDs (Tang et al., 2016). Previous reports indicate that presynaptic active zone proteins can form SSD structures in the absence of evoked release, in mice lacking presynaptic Ca_V_2 channels (Held et al., 2020), but it was unclear if active zone molecules still coalesce into SSDs in TeNT-expressing terminals lacking fusogenic vesicles. Thus, identical to previous experiments, we infected hippocampal neuronal cultures at DIV1 with our syph-GFP-IRES-TeNT virus. At DIV16, we used stimulated emission depletion microscopy (STED) to visualize pre-synaptic RIM1, along with post-synaptic GluA1. Both RIM1 and GluA1 SSDs could be clearly observed at control (Figure 3A), as well as at TeNT-expressing terminals (Figure 3B). The number of RIM1 SSDs was significantly increased in Syph-GFP-IRES-TeNT expressing terminals (Figure 3C). Interestingly individual SSDs were smaller, with reduced RIM1 signal intensity (Figures 3D,E). However, the overall summed RIM1 SSD area, per terminal was maintained (Figure 3F). Thus, TeNT does not impact the overall level of RIM1 but significantly influences its nanoscale distribution in the presynaptic terminal. Consistent with our dSTORM data, we observed no change in GluA1 SSD number, size, or summed area (per synapse) and further show that GluA1 signal intensity is not altered at TeNT-silenced synapses (Figures 3G–J). To assess if GluA1 was aligned with RIM1 at TeNT-silenced synapses, we quantified the fractional RIM1 SSD overlap of each GluA1 SSD. Here we constrained our overlap analyses to synapses viewed *en face* (Figure 3K). Surprisingly, we observed a small but significant increase in the percentage overlap of GluA1 SSDs with RIM1 SSDs, compared to controls (Figure 3K). Thus, AMPAR/RIM1 trans-synaptic alignment appears intact in TeNT-silenced terminals despite the fragmentation and redistribution of RIM1.

### Formation of inhibitory receptor and scaffold SSDs in the absence of neurotransmission

**Figure 4:**
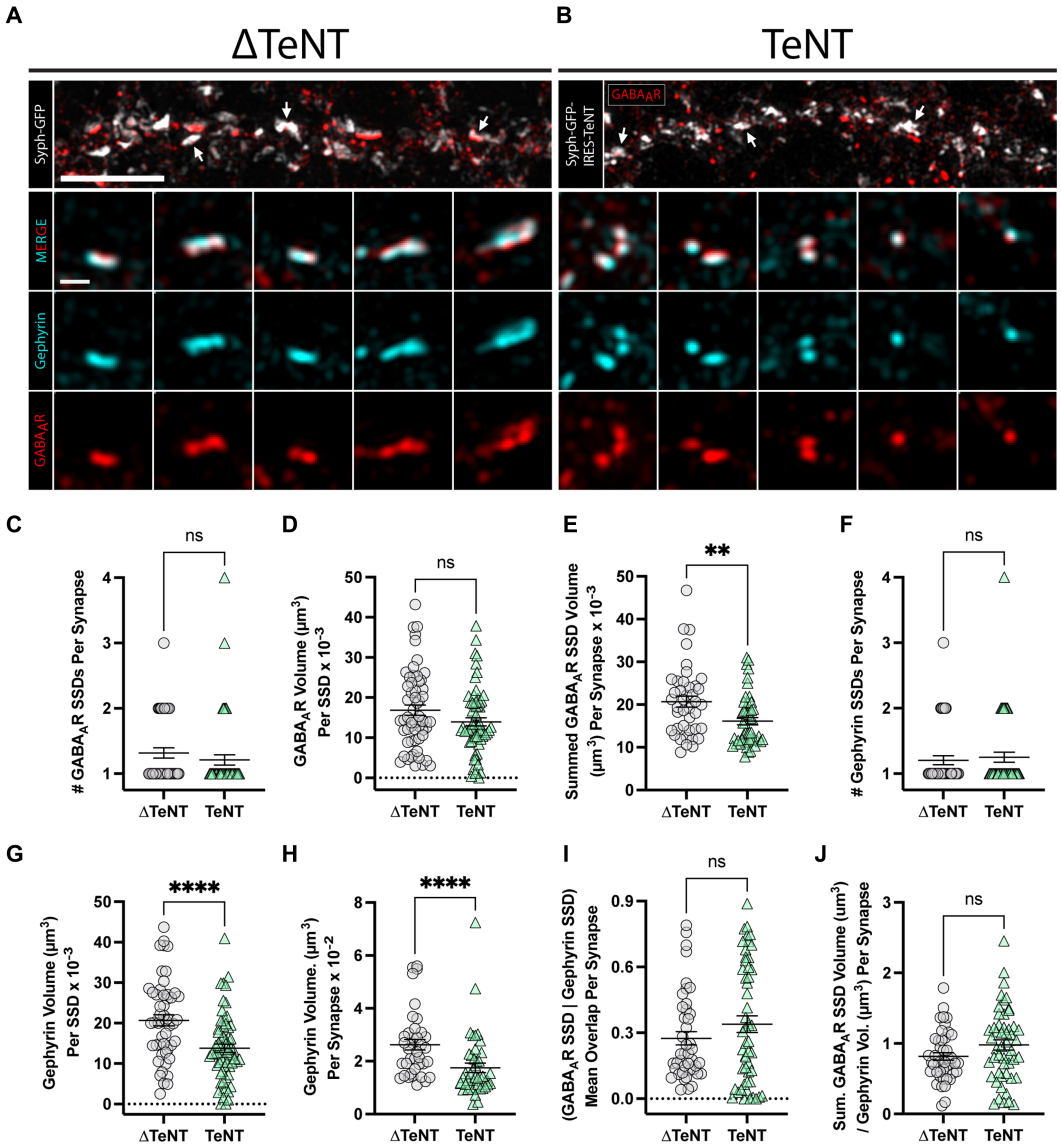
Formation of inhibitory receptor and scaffold SSDs in the absence of neurotransmission. **(A,B)** Representative SIM images of dendritic regions from (∆TeNT, **A**) or (TeNT, **B**), cultures displaying syph-GFP, or syph-GFP-IRES-TeNT (greyscale), and GABAAR (red). Note the apposition of syph-GFP signal and GABA_A_R-positive (red) synapses (white arrows). Scale bar = 5 μm. Columns 1–5 display representative synapses for ∆TeNT **(A)**, and TeNT **(B)** conditions; merged gephyrin (cyan), and GABA_A_R (red) (row 2); gephyrin (cyan) (row 3); and GABA_A_R (red) (row 4). Scale bar = 500 nm. **(C)** The number of GABA_A_R SSDs per synapse was not significantly different at TeNT-silenced synapses compared to controls [synapses, *n* = 44 ∆TeNT/52 TeNT; ns, not significant; *p* = 0.1340 (Mann–Whitney)]. **(D)** The volume of individual GABA_A_R SSDs was not significantly different at TeNT-silenced synapses compared to controls [SSDs, *n* = 57 ∆TeNT/63 TeNT; ns, not significant; *p* = 0.0739 (Mann–Whitney)]. **(E)** The summed synaptic volume of GABA_A_R SSDs per synapse was significantly decreased at TeNT-silenced spines compared to controls [synapses, *n* = 41 ∆TeNT/50 TeNT; **p < 0.01; *p* = 0.0020 (Mann–Whitney)]. **(F)** The number of gephyrin SSDs per synapse was not significantly different at TeNT-silenced synapses compared to controls [synapses, *n* = 44 ∆TeNT/52 TeNT; ns, not significant; *p* = 0.7200 (Mann–Whitney)]. **(G)** The volume of individual gephyrin SSDs was significantly reduced at TeNT-silenced synapses compared to controls [SSDs, *n* = 52 ∆TeNT/64 TeNT; ****p < 0.0001 (*T*-Test)]. **(H)** The total gephyrin volume per synapse was significantly decreased at TeNT-silenced synapses compared to controls [synapses, *n* = 38 ∆TeNT/44 TeNT; ****p < 0.0001 (Mann–Whitney)]. **(I)** There was no significant difference in the mean overlap between GABA_A_R SSDs and gephyrin SSD signals at TeNT-silenced synapses compared to controls [SSDs, *n* = 43 ∆TeNT/52 TeNT; ns, not significant; *p* = 0.5039 (Mann–Whitney)]. **(J)** The ratio between the summed synaptic volume of GABA_A_R SSDs, and the total gephyrin volume, per synapse was not significantly different at TeNT-silenced synapses compared to controls [synapses, *n* = 44 ∆TeNT/51 TeNT; ns, not significant; *p* = 0.0795 (*T*-Test)].

We used the same TeNT-based activity silencing strategy, along with three-dimensional, structured illumination microscopy (3D-SIM), to analyze the nanoscale architecture of the inhibitory synapse. Identical to our excitatory synapse experiments, hippocampal cultures were infected at DIV1 with lentivirus encoding either syph-GFP (viral control, ∆TeNT condition), or syph-GFP-IRES-TeNT (activity-silenced, TeNT condition). At DIV16, cultures were immunolabeled for surface GABA_A_Rs (γ2 subunit), fixed, permeabilized, and then immunolabeled for gephyrin, and imaged by 3D-SIM. We observed that both gephyrin, and GABA_A_Rs organized into SSDs at both viral control (Figure 4A), and chronically silenced synapses (Figure 4B). We observed no change to the number of GABA_A_R SSDs, per synapse, between conditions (Figure 4C), nor to the volume of individual GABA_A_R SSDs (Figure 4D). However, the summed GABA_A_R SSD volume, per synapse, was significantly decreased (Figure 4E) at chronically silenced synapses. This was likely due to the trending, but insignificant decreases in both the number of GABA_A_R SSDs per synapse (Figure 4C), and in the individual GABA_A_R SSD volume (Figure 4D). We observed no change to the number of gephyrin SSDs per synapse (Figure 4F). However, individual gephyrin SSDs, were dramatically reduced in volume (Figure 4G) at chronically silenced synapses, and the overall gephyrin volume was also significantly smaller, per synapse, indicating reduced overall size of the postsynaptic scaffold network (Figure 4H). The summed GABA_A_R SSD volume decreased in proportion with total gephyrin volume, at individual synapses (Figure 4J). Lastly, the average per-synapse overlap between GABA_A_R SSDs, and gephyrin SSDs was unaffected (Figure 4I). Together, these data show that while neurotransmission is dispensable for GABA_A_R and gephyrin SSD formation/colocalization, it plays a major role in expanding the postsynaptic inhibitory scaffolding network.

## Discussion

Here we tested whether synaptic neurotransmitter release was required at any point during synapse development for the formation of sub-synaptic nano-architecture. Previous work shows that scaffold SSDs are maintained and can develop in response to activity blockade at established synapses (MacGillavry et al., 2013; Sun et al., 2022). Our experiments extend these observations by demonstrating that neurotransmitter signaling prior to, and throughout, synaptic development is dispensable for the establishment of both scaffold, and receptor SSDs. Our work also complements prior studies investigating the role of activity on synapse development, and nano-architecture. For instance, neuronal dendrite development (Harms and Craig, 2005), and synapse formation (Verhage et al., 2000; Varoqueaux et al., 2002; Harms et al., 2005; Harms and Craig, 2005; Sando et al., 2017; Sigler et al., 2017; Hazan and Ziv, 2020) occur independently of synaptic transmission. A recent study investigating the developmental refinement of excitatory synaptic SSDs found that blocking action potentials during synapse maturation with TTX had only a modest impact on PSD95 SSD properties and had no effect on trans-synaptic PSD95/RIM1 alignment (Sun et al., 2022). However, in these experiments, it is possible that ongoing spontaneous miniature release could be responsible for the formation/maintenance of synaptic nano-architecture. Our experiments rule out this possibility since TeNT blocks both evoked and spontaneous synaptic release. However, it remains possible that tonic, non-synaptic neurotransmitter release could mediate some aspects of synaptic nanoarchitecture development (Verderio et al., 1999).

How do receptor and scaffold SSDs form to begin with? Recent data support roles for adhesion molecules not only for synapse formation, but also for maintenance of subsynaptic AMPAR clusters and their trans-synaptic alignment with presynaptic neurotransmitter release sites (Biederer et al., 2017). For example, presynaptic neurexin-3 appears to coordinate recruitment of postsynaptic AMPARs (Aoto et al., 2013). Acute disruption of the postsynaptic adhesion molecule LRRTM2 led to fewer and smaller AMPAR clusters suggesting it could also be an important organizing molecule nucleating AMPAR nanodomains (Ramsey et al., 2021). At inhibitory synapses, NL2 is also found to form SSDs and could contribute to GABA_A_R nanoscale organization and nanocolumn formation (Gookin et al., 2022). Additionally, recent evidence suggests postsynaptic proteins could spontaneously segregate into subsynaptic structures based on the physical principles of phase separation. *In vitro* studies using purified proteins show that the C-terminus of the AMPAR auxiliary subunit stargazin condenses into a phase separated structure when mixed with PSD95, albeit on a much different spatial scale than the nanodomains observed at synapses (Zeng et al., 2019; Hosokawa et al., 2021). Nevertheless, subsynaptic condensation of specific collections of PSD proteins is an attractive mechanism for activity-independent formation of subsynaptic molecular nanoarchitecture.

While receptor and scaffold SSDs can form at chronically silenced synapses, their properties were significantly different compared to control, active synapses. For example, we observed decreased number of PSD95 SSDs when neurotransmitter release was chronically blocked at excitatory synapses. This resulted in reduced overall area of the excitatory postsynaptic compartment, consistent with previous studies demonstrating fewer AMPARs and reduced amplitude of uncaging-evoked postsynaptic AMPAR currents at synapses lacking presynaptic release machinery (Ehlers et al., 2007; Sigler et al., 2017). On the other hand, numerous previous studies show that activity blockade at established synapses leads to larger PSDs and increased AMPAR levels (Hou et al., 2008; Béïque et al., 2010; MacGillavry et al., 2013; Dubes et al., 2022). However, in these studies, activity was blocked following synaptogenesis for a relatively short time period (2–3 days), while in our experiments TeNT-silenced synapses have likely never experienced evoked or spontaneous neurotransmission. Thus, neurotransmitter signaling at some point during development could be required to prime synapses for subsequent homeostatic alterations. Our results are consistent with the lack of homeostatic modifications to overall synaptic AMPAR levels observed when purified TeNT was exogenously applied prior to and throughout synapse formation and maturation (Harms and Craig, 2005). In contrast to the apparently smaller PSDs we observed, another study using mice expressing TeNT in excitatory principal neurons reported slightly increased PSD volumes at mushroom-type spines, measured by 3D electron microscopy (EM) (Sando et al., 2017; Zhu et al., 2021). However, in agreement with our results, PSD volume was reduced at stubby, thin and filopodial spines (Zhu et al., 2021). Unfortunately, we could not classify spine morphology in our data sets due to limitations in the number of available imaging channels. It is also possible that in our experiments we are failing to capture the entire PSD volume using a single molecular scaffold (PSD95) as a readout. Potential activity-dependent expression and sub-PSD positioning of diverse excitatory and inhibitory scaffolds as well as other AMPAR subunits will be interesting to investigate in future work.

Surprisingly, blocking vesicle fusion with TeNT did not prevent RIM1 from assembling into subsynaptic structures, nor the trans-synaptic alignment of RIM1 SSDs with post-synaptic AMPARs. Similar results were observed in a different study where neurotransmission was blocked by knockout of presynaptic Ca^2+^ channels, with no impact on trans-synaptic alignment (Held et al., 2020). However, in contrast to that study, we observed an increased number of smaller RIM1 SSDs at TeNT-silenced synapses. These changes were not reciprocated at the postsynaptic membrane, as the number of AMPAR SSDs per synapse was not significantly altered. Thus, at TeNT-silenced synapses, there were more “orphan” RIM1 SSDs, suggesting that RIM1 does not dictate AMPAR nano-localization, at least under these conditions. It is possible that RIM1 SSDs lacking apposed AMPAR SSDs are mislocalized and do not label active zone structures, given the lack of fusion-competent vesicles. On the other hand, TeNT treatment leads to an overabundance of docked vesicles (Schiavo et al., 2000), consistent with our observations of RIM1 being more widely distributed in TeNT-expressing terminals.

At chronically silenced inhibitory synapses we observed similar changes in postsynaptic size and molecular nano-architecture. For example, we measured a reduction in the total gephyrin volume per synapse, which was driven by decreased volume of individual gephyrin SSDs. In addition, we also observed a reduction in the summed synaptic GABA_A_R SSD volume at silenced synapses. Thus, while synaptic transmission is not required for SSD formation, it could play a role in recruiting additional gephyrin to enlarge and maintain existing scaffold subnetworks. Future experiments may reveal additional alterations to the number, size and placement of gephyrin/GABA_A_R SSDs relative to presynaptic active zones. Whether changes in overall inhibitory synapse size and gephyrin SSD measurements are a direct result of input-specific blockade of GABA release, or a compensatory response to reduced excitatory neurotransmission in TeNT-infected cultures remains to be seen. Future experiments sparsely silencing and labeling GABAergic inputs from specific interneuron populations will be required to assess any direct role for GABA transmission in defining inhibitory nano-architecture, which could be especially relevant during the developmental time window during which GABA is excitatory.

## Materials and methods

### Neuronal cultures

Animal procedures for all experiments herein were conducted in accordance with the University of Colorado, Anschutz Medical Campus, Institutional Animal Care and Use Committee (IACUC). Hippocampal neuronal cultures were prepared from postnatal P0-P1 day old male and female Sprague–Dawley rat pups (Charles River Laboratories). Hippocampi were first dissociated via papain, and cells were then plated in MEM (supplemented with 10% FBS, 50 U/mL penicillin/streptomycin, and L-glutamine) on 18 mm glass coverslips coated with poly-D lysine at a density of 125,000–150,000 cells per coverslip. On the following day, cell media was replaced with Neurobasal-A (NBA) media (supplemented with B27, and GlutaMAX). For all experiments, Lentivirus (encoding syph-GFP or syph-GFP-IRES-TeNT) was added to cultures on DIV1 directly following the media change. Uninfected controls received no virus. Mitotic inhibitors (uridine, and 5-Fluoro-2` deoxyridine) were added to NBA media, which was used to replace half of the well media, in each well, on DIV7. Experimental timepoints prior to, and including DIV7 did not receive anti-mitotic treatment.

### VAMP2 immunocytochemistry

#### Immunocytochemistry

Coverslips were rinsed once with warmed 1X PBS, and cells were then fixed with 4% PFA, for 15 min. Following fixation, coverslips were rinsed again in 1X PBS. Cells were then permeabilized, with 0.1% PBS-Triton (PBST), for 10 min, at slow rotation. A 15 min blocking step was then performed using 5% bovine serum albumin (BSA), at slow rotation. Primary antibody solution (PABS) was made using 5% BSA and included anti-bassoon (1:500) (Synaptic Systems, 141,004), and anti-synaptobrevin 2 (1:1000) (Synaptic Systems, 104,211). PABS was added to each well for a 30 min incubation, at slow rotation. Following PABS incubation, coverslips were rinsed thrice in 1X PBS. Secondary antibody solution (SABS) was made using 5% BSA, and included Alexa-Fluor (AF) 568, and 647 (1:1000). SABS was added to each well for a 30 min, light-protected incubation, at slow rotation. Following SABS incubation, coverslips were again rinsed thrice in 1X PBS. Cells were then post-fixed with 4% PFA for a 5 min, light-protected incubation, at slow rotation, followed by three additional rinses in 1X PBS. Coverslips were then mounted to glass microscope slides, using 10 uL mounting media, per coverslip. ICC for each of the five experimental timepoints were performed identically.

#### Confocal image acquisition

Coverslips were imaged on an Olympus IX71 equipped with a spinning-disc scan head (Yokogawa). Excitation illumination was delivered from an acousto-optic tunable filter (AOTF) controlled laser launch (Andor). Images were acquired using a 60x Plan Apochromat 1.4 numerical aperture objective and collected on a 1,024 × 1,024 pixel Andor iXon EM-CCD camera. 2–3 μm z-stacks were imaged with a 0.2 μm step size. Laser power, and exposure times were varied between experimental timepoints as needed, but were kept identical between control and experimental conditions.

#### Data analysis

First, a maximum projection image was created from each Z-stacked region. The VAMP2 channel’s acquisition image was then created as a separate, maximum projection image, and placed aside. With the circle ROI selector set to a size of 10 pixels, a wide-field grid pattern was overlaid on the max-composite, multi-channel image, to assist in ROI selection. ROIs were then selected using FIJI’s ROI manager tool. For control images, single punctate synaptic terminals were selected, based solely on bassoon immunofluorescence. For TeNT images, single punctate synaptic terminals were selected, based solely on syph-GFP-IRES-TeNT immunofluorescence. To avoid bias, VAMP2 immunofluorescence was not visualized prior to the selection of control or TeNT ROIs. After selecting synaptic ROIs, four background ROIs were selected based on the absence of fluorescence in each channel, and were selected from the top, left, right, and bottom quadrants of the image. The VAMP2 maximum projection was then selected, and the ROI manager’s ‘show all’ option was used to impart all chosen ROIs to the image. The ‘measure’ option in ROI manager was then used to calculate the ROI area, and integrated density. Mean background fluorescence and corrected total terminal fluorescence (CTTF) were then calculated; mean background fluorescence for a given image was calculated by averaging the four background ROIs’ mean gray values; VAMP2 CTTF was then calculated for each ROI/terminal [ROI VAMP2 integrated density – (ROI area x mean background fluorescence)].

### Synaptic FM Dye loading

#### FM Dye loading

Coverslips were first incubated in ACSF containing (in mM) 130 NaCl, 5 KCl, 10 HEPES, 11 glucose, 2 CaCl_2_, 1 MgCl_2_, 0.01 NBQX, 0.1 DL-APV, 0.01 FM4-64FX for 2 min (pre/post-stimulation solution). Solution was then removed, and stimulation solution (isosmotic ACSF with 50 mM KCl plus 10 μM NBQX, 100 μM DL-APV, 10 μM FM4-64FX) was then applied for 1 min. Solution was then removed, and coverslips were incubated in pre/post-stimulation solution for 5 min. Solution was then removed and washout solution (ACSF, 10 μM NBQX, 100 μM DL-APV) was applied for 30 s, and repeated for 3 cycles. Samples were then rinsed once with 1X PBS. Finally, coverslips were incubated in 4% PFA, for 5 min, followed by three rinses of 1X PBS. Immunocytochemistry was then performed, beginning with a permeabilization step, via 0.1% PBST, for 10 min, at slow rotation. Permeabilization was followed by a blocking step, via 5% BSA, for 15 min, at slow rotation. PABS was then created with 5% BSA, and included anti-bassoon (1:500) (Synaptic Systems, 141,004) for control samples; samples underwent a 30 min incubation, at slow rotation, followed by three rinses with 1X PBS. SABS was then made in 5% BSA, and included DyLite 405 (1:1000) (Invitrogen, PISA510094); samples underwent a 30 min incubation, at slow rotation, followed by three rinses with 1X PBS. Samples were then post-fixed, via 4% PFA, for 5 min, followed by three final rinses with 1X PBS, before mounting to glass microscope slides, using 10 uL mounting media, per coverslip. Immunocytochemistry for each of the five experimental timepoints were performed identically. Images were acquired as described above. FM4-64 quantification proceeded nearly identically to those of the VAMP2 staining experiments, except that here, immunofluorescence quantification was performed on the FM dye signal. Once again, ROIs for the TeNT condition were chosen solely based on syph-GFP-IRES-TeNT terminal immunofluorescence without visualizing the FM4-64 channel to avoid selection bias. The number of TeNT ROIs was then set as the target number of ROIs for control ROI selection, with control ROIs selected solely based on bassoon terminal fluorescence to avoid selection bias.

### Electrophysiology

mEPSCs were measured using whole cell voltage clamp at −70 mV from uninfected control cultures, and from syph-GFP-IRES-TeNT expressing cultures. 2 min gap free recordings were made ~2 min following break in. mEPSCs were quantified using MiniAnalysis software (Synaptosoft Inc). aCSF solution (in mM) contained: 10 HEPES, 130 NaCl, 30 D-glucose, 5 KCL, 0.002 TTX, 0.02 Bicuculline. Internal recording solution (in mM): 130 CsMeSO4, 2.5 NaCl, 5 MgCl2, 10 HEPES, 0.5 EGTA, 0.5 Na3GTP, 3 Na2ATP, 10 phosphocreatine (290–300 mOsm). The pH was adjusted to 7.25 with CsOH.

### PSD95/AMPAR dSTORM imaging

#### Immunocytochemistry

Neurons were first surface-labeled for the AMPAR subunit, GluA1 using a custom made antibody (Sinnen et al., 2017) against the extracellular N-terminus added directly to the tissue culture well at 1:300 dilution for a 15 min live-incubation. Cells were then fixed via 15 min incubation in 4% PFA. Samples were then rinsed thrice in 1X PBS, permeabilized for 10 min in 0.1% PBST, and blocked for 30 min in 5% BSA. A second PABS was then made in 5% BSA, and included anti-PSD95 (1:1000) (Abcam, ab2723), and was added to coverslips for overnight incubation. On the following day, coverslips were first rinsed three times in 1X PBS. SABS was then made in 5% BSA, and included CF568 (1:1000), and AF647 (1:1000). Coverslips underwent a 2 h, light-protected incubation, followed by three rinses with 1X PBS. A 15 min post-fixation step was then performed, via 4% PFA, followed by three final 1X PBS rinses. Coverslips were then returned to a new 12-well plate, with each coverslip well receiving 2 mL fresh 1X PBS. The plate was then parafilm sealed, and light-protected, until dSTORM imaging.

#### Image acquisition

Most samples were imaged within several days of, but no later than 2 weeks following ICC. In preparation for each imaging session, a 1:1000 dilution of 0.1 μm fluorescent beads was diluted in 0.5X PBS, which would be used in channel calibration acquisitions taken prior to sample imaging. For each sample coverslip, fresh dSTORM buffer (TRIS buffer, Cysteamine hydrochloride (MEA), glucose oxidase (GLOX)) was prepared, with pH adjusted to 8.5, by addition of 5 N KOH. Buffer was then filtered through a 0.2 micron syringe filter attached to a 3 mL syringe, onto the respective coverslip, which was situated in a sealed imaging chamber, atop which a second coverslip was placed to preserve buffer integrity throughout the course of imaging.

dSTORM imaging was performed on a Zeiss Elyra P1 microscope, equipped with 4 excitation laser lines (405 nm/50 mW, 488 nm/200 mW, 561 nm/150 mW, 642 nm/200 mW) and an Andor iXon+897 with a back illuminated 512 × 512 EMCCD chip. A Zeiss alpha Plan Apochromat TIRF 100X/1.46NA oil objective (Zeiss # 420792–9,800-720) was used for dSTORM acquisitions which in conjunction with the tube lens, and an additional 1.6x magnification lens yielded a pixel size of 100 nm in the raw data.

When selecting imaging regions, a 10×10, 3-channel tile scan was typically used to find optimal sample regions; we sought to locate and image dendrites from uninfected cells (i.e., lacking detectable somal, and dendritic syph-GFP fluorescence). Thus, cells in which syph-GFP strongly fluoresced within their soma or dendritic compartments were avoided. Each selected region was then imaged under epifluorescence prior to STORM acquisition. Imaging parameters were then changed to perform STORM imaging on the region, with time series of 20,000 frames to be acquired for each channel, 647, and 561, sequentially.

Samples were illuminated in ultra high power, HiLo mode, which resulted in a usable image area of 256 × 256 pixels (25.6 μm x 25.6 μm). Separate filter cubes were used for imaging AF 647 and *CF* 568 dyes, containing a long pass 655 nm filter and a 570 nm – 650 nm band pass filter, respectively, for fluorescence emission selection. An additional 405/488/561/642 nm notch filter was placed in front of the camera to reject laser light. Ground state depletion in both channels was accomplished by 100% relative excitation power which resulted in power densities of around 1.4 W/cm^2^ for the Alexa 647, and around 2.5 W/cm^2^ for the *CF* 568 channel. In both imaging channels, the return to ground state was stimulated by continuous illumination with the 405 nm laser at relative intensities between 0.1 and 5%.

#### Initial processing

For specific details on data processing, see relevant sections within Gookin et al., 2022. In brief, the Bio-Formats MATLAB toolbox (Linkert et al., 2010) enabled MATLAB to read raw data files exported from Zeiss. Non-homogenous background was first removed via temporal filtering (Hoogendoorn et al., 2014). The ThunderSTORM ImageJ plugin (Ovesný et al., 2014) was used to localize emitting dyes. This was followed by applying a temporal median filter, image filtering, and an approximate first-pass molecular localization, based on the point spread function (PSF). Localizations were then filtered further, based on several attributes, including uncertainty, sigma, and intensity. The set of 10 bead calibration images acquired prior to the experiment were compiled into a single set, which was used to correct for distortions between the 642 and 561 channels. Bead positions were fitted between channels, and shifts in both the x and y directions were calculated across the entire field of view. By applying this calibration to our STORM data, the between-channel distortion, which can exist up to 100 nm, is reduced to an RMS error of <15 nm of misalignment between channels. An additional drift correction was then performed (Wang et al., 2014).

#### ROI analysis

For specific details on data analysis, see relevant sections within Gookin et al., 2022. In summary, our dSTORM coordinate analysis is based on previous methods used to analyze nanoscale substructure (Tang et al., 2016). Synapses were chosen based on the colocalized fluorescence of either syph-GFP (∆TeNT), or syph-GFP-IRES-TeNT (TeNT) pre-synaptic terminals, and PSD95/GluA1 immunofluorescent post-synaptic dendritic spines, in the epifluorescence image. For choice in analysis, we required that selected spines were undoubtedly contacted (either by ~50% terminal/spine overlap, or by unambiguous, and direct adjacency) by their nearby infected terminal (i.e., spines too close to neighboring spines, which decrease certainty of which spine is contacted by the terminal, were not chosen), and that the spine borders could be clearly defined within the STORM image. ROIs were then chosen in the STORM image, for further analysis. For a given ROI, the ROI-specific density range was used to calculate thresholding parameter; this circumvented issues which might arise from differences in labeling density of sample regions. Localizations within the lower 10% of the respective ROI’s local density range were not classified as within the synaptic region/SSD. Each region’s boundaries were determined using the alphaShape function within MATLAB, with a set alpha value of 100. High density regions, or HDRs, were delineated via randomization of the measured experimental localizations, under the assumptions of distribution uniformity within the synaptic region. In order for a localization to be classified as part of an HDR, its local density measurement needed to fit within the randomized dataset’s mean local density, plus 2 standard deviations. The MATLAB alphaShape function was used once more to determine the geometric boundaries of each HDR, but here an alpha value of 7 was used.

### RIM1/AMPAR STED imaging

#### Immunocytochemistry

Samples were first live-labeled against the GluA1 subunit of the AMPAR; anti-GluA1 IgG (1:100) (Andrews et al., 2019), was added for a 30 min incubation, at 37°C. Following GluA1 labeling, coverslips were rinsed thrice with warmed aCSF, and fixed with 2% PFA, containing 4% sucrose, for 10 min. Samples were then washed three times, each for 5 min, with 1X PBS and glycine, for a total of 15 min, at slow rotation. Samples were then permeabilized for 20 min at slow rotation, in 0.1% TBST. Next, samples were blocked using 5% goat serum and 5% BSA, for 20 min, at slow rotation. Finally, a second PABS which contained anti-RIM1 (1:500, Synaptic Systems, 140,003, LOT#7–30), was added to samples, which were left to incubate overnight, at 4°C. On the following day, samples were first washed three times, each for 5 min, with 1X PBS and glycine, for a total of 15 min, at slow rotation. SABS was then made, and included STAR 580/635P (1:500, Abberior, LOT20420PK-4 and 21007PK-2, respectively), in which samples underwent a light-protected incubation, for 2 h, at room temperature. Samples were then washed three times, each for 5 min, with 1X PBS and glycine, for a total of 15 min, at slow rotation, followed by a post-fixation step using 2% PFA/4% sucrose, for 20 min, at room temperature. Samples were then washed three final times, each for 5 min, in 1X PBS and glycine, at slow rotation, before mounting each coverslip to a glass microscope slide with 10 μL of mounting media.

#### Image acquisition

STED images of the Abberior STAR 635 and Abberior STAR 580 channels were acquired with an Abberior STEDYCON in the Anschutz Medical Campus Advanced Light Microscopy Core (ALMC) facility, which is installed on an Olympus IX81 microscope stand, using a 100X/1.45NA Olympus UPLXAPO100XO oil immersion objective. Sample regions were first identified by eye in the TRITC epifluorescence channel by selecting parts of the sample with strong punctate labeling in the Abberior STAR 580 channel. Following this, a 5 × 5 tiled overview image was acquired in confocal mode and the regions with even labeling, and the lowest background were selected for STED imaging. ROIs were selected based on the orientation of dendritic branches, due to the nature of the scanning device, which enables faster and more efficient acquisitions, meaning wide rectangles were used about the height of a laterally protruding dendritic branch. Pixel size was 25 nm. Laser power was first established such that a count of ~100 photons per pixel in confocal mode was established, then STED laser power was set to about 3-fold the laser power in confocal mode. Once determined, these laser intensities remained the same across all conditions.

#### STED analysis

En face synaptic ROIs were selected using a custom routine in Fiji (ImageJ 2.90/1.53 t, Java version 1.8.0_322, Rasband, W., et al., National Institute of Health), which allows the user to draw a box around an individual synapse and save out the ROI coordinates and a multi-channel TIFF. Only puncta that were positive for Syph-GFP were selected in the TeNT condition. The extracted TIFFs were segmented using the Squassh function in MosaicJ software, in Fiji. Folder structure to permit analysis was performed with custom routines in MATLAB (MathWorks, 2023). The segmented data contained a pixel size of 25 nm, which permitted area conversions and the collection of their intensities, which were exported to GraphPad Prism (Version 8.0.0 for Windows, GraphPad Software, San Diego, California, United States), where statistical analyses were performed.

### Gephyrin/GABA_A_R SIM imaging

#### Immunocytochemistry

Neurons were fixed in 4% PFA solution (1X PBS, 4% sucrose, and 50 mM HEPES (pH7.5)) for 5 min, at room temperature, followed by three washes with 1X PBS. Sample coverslips were then incubated in blocking solution (1X PBS, 5% BSA, 2% Normal Goat Serum (NGS)), at room temperature, for 1 h. Surface labeling against the GABA_A_R, γ2-subunit (1:500) (Synaptic Systems, 224–004) was performed, under nonpermeabilized conditions, in blocking solution, for 1 h, at room temperature. Neurons were then washed three times in 1X PBS, followed by permeabilization in 0.5% NP-40 for 2 min, and blocked at room temperature for 30 min. Labeling against gephyrin, 3B11 (1:500) (Synaptic Systems, 147,111) was performed in blocking solution, for 1 h. Neurons were then washed three times in 1X PBS, and then labeled with secondary antibodies Alexa Fluor 568, and 647 (1:1000) (ThermoFisher), for 1 h at room temperature. Coverslips were then washed four times in 1X PBS and mounting media was used to adhere the slips to glass microscope slides.

#### Image acquisition

3D-SIM images were acquired using a Structured Illumination super-resolution microscope (Nikon SIM-E, M645E). A 100X, 1.49 NA objective was used, and images were captured with an ORCA-Flash 4.0 sCMOS camera (Hamamatsu). To maximize signal-to-noise, and to reduce photobleaching, acquisition conditions and camera integration time were set in a similar manner to (Crosby et al., 2019). Synapses were captured in entirety within the Z-stack.

#### ROI selection, and data analysis

Following selection of synaptic ROIs, ROIs were processed by background subtraction (ImageJ), image segmentation (split Bregman/MOSAIC suite) (Rizk et al., 2014), and geometric analysis (MATLAB), as further detailed in (Crosby et al., 2019). For image segmentation, the following parameters were utilized: “Subpixel segmentation,” “Exclude Z edge,” Local intensity estimation “Medium,” Noise Model “Gauss.” All 3D-SIM imaging was analyzed blind to experimental condition.

### Statistical analyses

Statistical analyses were performed in Graphpad Prism (version 9.4.0 (453)). Each analysis was performed as described. In most cases, the ROUT method of outlier analyses was performed, with a default value of 1% aggression; when outliers were detected, cleaned data excluding those values were used for further analyses (in the case of data from the VAMP2 terminal fluorescence, and FM dye terminal fluorescence experiments, outlier analyses were not utilized due to large sample size). Following outlier analyses, values from each condition were then plotted in histogram form, to allow for a visual assessment of normality. Next, formal normality tests (D’Agostino-Pearson, Anderson-Darling, Shapiro–Wilk, and Kolmogorov–Smirnov) were run to either corroborate, or deny visual assessments of normality. Homoscedasticity was measured by dividing the larger standard deviation value, by the smaller standard deviation value, with a value of 2.00 or less being used as the cutoff for which data was considered of equal variance. Finally, a decision on the most appropriate statistical test was made; in most cases, when data from either one, or both conditions failed to meet normality, but where data did meet the homoscedasticity requirement, a Mann–Whitney test was used, in lieu of a standard T-Test. In most cases, where groups failed to meet normality, but the homoscedasticity requirement was also unmet, a T-Test with Welch’s correction was used.

## Materials and reagents

Please direct all inquiries regarding materials, and/or reagents used in our experiments to corresponding author, MK (matthew.kennedy@cuanschutz.edu).

## Data availability statement

The raw data supporting the conclusions of this article will be made available by the authors, without undue reservation.

## Ethics statement

The animal study was reviewed and approved by the University of Colorado Anschutz Medical Campus Institutional Animal Care and Use Committee.

## Author contributions

HR performed the VAMP2 immunofluorescence time-course, FM dye loading time-course, and PSD95/AMPAR dSTORM experiments, and all respective analyses. DK performed the electrophysiology and analysis. AR performed the RIM1/AMPAR STED experiments and analyses. SG performed the Gephyrin/GABAAR SIM experiments and analysis. DS provided the technical support for STED and dSTORM microscopy. HR and MK prepared the manuscript. KS and MK provided experimental design, funding, and supervision. All authors contributed to the article and approved the submitted version.

## Funding

This work was supported by the T32 NS099042 Neuroscience Predoctoral Training Grant, (HR); R35NS116879, UF1NS107710 (MK); R01MH119154 (KS); F31NS130979 (DK).

## Conflict of interest

The authors declare that the research was conducted in the absence of any commercial or financial relationships that could be construed as a potential conflict of interest.

## Publisher’s note

All claims expressed in this article are solely those of the authors and do not necessarily represent those of their affiliated organizations, or those of the publisher, the editors and the reviewers. Any product that may be evaluated in this article, or claim that may be made by its manufacturer, is not guaranteed or endorsed by the publisher.

## References

[ref1] Andrews N. P. Boeckman J. X. Manning C. F. Nguyen J. T. Bechtold H. Dumitras C.. (2019). A toolbox of IGG subclass-switched recombinant monoclonal antibodies for enhanced multiplex immunolabeling of brain. eLife 8. doi: 10.7554/elife.43322 PMC6377228 30667360

[ref2] Aoto J. Martinelli D. C. Malenka R. C. Tabuchi K. Südhof T. C. (2013). Presynaptic neurexin-3 alternative splicing trans-synaptically controls postsynaptic AMPA receptor trafficking. Cell 154, 75–88. doi: 10.1016/j.cell.2013.05.060, PMID: 23827676 PMC3756801

[ref3] Béïque J.-C. Na Y. Kuhl D. Worley P. F. Huganir R. L. (2010). ARC-dependent synapse-specific homeostatic plasticity. Proc. Natl. Acad. Sci. 108, 816–821. doi: 10.1073/pnas.1017914108, PMID: 21187403 PMC3021034

[ref4] Betz W. J. Bewick G. S. (1992). Optical analysis of synaptic vesicle recycling at the frog neuromuscular junction. Science 255, 200–203. doi: 10.1126/science.1553547, PMID: 1553547

[ref5] Biederer T. Kaeser P. S. Blanpied T. A. (2017). Transcellular nanoalignment of synaptic function. Neuron 96, 680–696. doi: 10.1016/j.neuron.2017.10.006, PMID: 29096080 PMC5777221

[ref7] Chen H. Tang A.-H. Blanpied T. A. (2018). Subsynaptic spatial organization as a regulator of synaptic strength and plasticity. Curr. Opin. Neurobiol. 51, 147–153. doi: 10.1016/j.conb.2018.05.004 29902592 PMC6295321

[ref8] Crosby K. C. Gookin S. E. Garcia J. D. Hahm K. M. Dell’Acqua M. L. Smith K. R. (2019). Nanoscale subsynaptic domains underlie the organization of the inhibitory synapse. Cell Rep. 26, 3284–3297.e3. doi: 10.1016/j.celrep.2019.02.070, PMID: 30893601 PMC6529211

[ref9] Dubes S. Soula A. Benquet S. Tessier B. Poujol C. Favereaux A.. (2022). Mir-124-dependent tagging of synapses by synaptopodin enables input-specific homeostatic plasticity. EMBO J. 41:e109012. doi: 10.15252/embj.2021109012, PMID: 35875872 PMC9574720

[ref10] Ehlers M. D. Heine M. Groc L. Lee M.-C. Choquet D. (2007). Diffusional trapping of GluR1 AMPA receptors by input-specific synaptic activity. Neuron 54, 447–460. doi: 10.1016/j.neuron.2007.04.010, PMID: 17481397 PMC1993808

[ref11] Fukata Y. Dimitrov A. Boncompain G. Vielemeyer O. Perez F. Fukata M. (2013). Local palmitoylation cycles define activity-regulated postsynaptic subdomains. J. Cell Biol. 202, 145–161. doi: 10.1083/jcb.201302071, PMID: 23836932 PMC3704990

[ref12] Goncalves J. Bartol T. M. Camus C. Levet F. Menegolla A. P. Sejnowski T. J.. (2020). Nanoscale co-organization and coactivation of Ampar, NMDAR, and mglur at excitatory synapses. Proc. Natl. Acad. Sci. 117, 14503–14511. doi: 10.1073/pnas.1922563117, PMID: 32513712 PMC7321977

[ref13] Gookin S. E. Taylor M. R. Schwartz S. L. Kennedy M. J. Dell’Acqua M. L. Crosby K. C.. (2022). Complementary use of super-resolution imaging modalities to study the nanoscale architecture of inhibitory synapses. Front. Syn. Neurosci. 14:2227. doi: 10.3389/fnsyn.2022.852227, PMID: 35463850 PMC9024107

[ref14] Haas K. T. Compans B. Letellier M. Bartol T. M. Grillo-Bosch D. Sejnowski T. J.. (2018). Pre-post synaptic alignment through neuroligin-1 tunes synaptic transmission efficiency. elife 7. doi: 10.7554/elife.31755, PMID: 30044218 PMC6070337

[ref15] Harms K. J. Craig A. M. (2005). Synapse composition and organization following chronic activity blockade in cultured hippocampal neurons. J. Comp. Neurol. 490, 72–84. doi: 10.1002/cne.20635, PMID: 16041714

[ref16] Harms K. J. Tovar K. R. Craig A. M. (2005). Synapse-specific regulation of AMPA receptor subunit composition by activity. J. Neurosci. 25, 6379–6388. doi: 10.1523/jneurosci.0302-05.2005, PMID: 16000628 PMC6725282

[ref17] Hazan L. Ziv N. E. (2020). Activity dependent and independent determinants of synaptic size diversity. J. Neurosci. 40, 2828–2848. doi: 10.1523/jneurosci.2181-19.2020, PMID: 32127494 PMC7117895

[ref18] Heilemann M. van de Linde S. Schüttpelz M. Kasper R. Seefeldt B. Mukherjee A.. (2008). Subdiffraction-resolution fluorescence imaging with conventional fluorescent probes. Angew. Chem. Int. Ed. 47, 6172–6176. doi: 10.1002/anie.200802376 18646237

[ref19] Held R. G. Liu C. Ma K. Ramsey A. M. Tarr T. B. De Nola G.. (2020). Synapse and active zone assembly in the absence of presynaptic ca2+ channels and ca2+ entry. Neuron 107, 667–683.e9. doi: 10.1016/j.neuron.2020.05.032, PMID: 32616470 PMC7442750

[ref20] Hoogendoorn E. Crosby K. C. Leyton-Puig D. Breedijk R. M. Jalink K. Gadella T. W.. (2014). The fidelity of stochastic single-molecule super-resolution reconstructions critically depends upon robust background estimation. Sci. Rep. 4. doi: 10.1038/srep03854, PMID: 24458236 PMC3900998

[ref21] Hosokawa T. Liu P.-W. Cai Q. Ferreira J. S. Levet F. Butler C.. (2021). CaMKII activation persistently segregates postsynaptic proteins via liquid phase separation. Nat. Neurosci. 24, 777–785. doi: 10.1038/s41593-021-00843-3, PMID: 33927400

[ref22] Hou Q. Zhang D. Jarzylo L. Huganir R. L. Man H.-Y. (2008). Homeostatic regulation of AMPA receptor expression at single hippocampal synapses. Proc. Natl. Acad. Sci. 105, 775–780. doi: 10.1073/pnas.0706447105, PMID: 18174334 PMC2206612

[ref23] Hruska M. Cain R. E. Dalva M. B. (2022). Nanoscale rules governing the organization of glutamate receptors in spine synapses are subunit specific. Nat. Commun. 13:920. doi: 10.1038/s41467-022-28504-4, PMID: 35177616 PMC8854560

[ref24] Hruska M. Henderson N. Le Marchand S. J. Jafri H. Dalva M. B. (2018). Synaptic nanomodules underlie the organization and plasticity of spine synapses. Nat. Neurosci. 21, 671–682. doi: 10.1038/s41593-018-0138-9, PMID: 29686261 PMC5920789

[ref25] Lee S. H. Jin C. Cai E. Ge P. Ishitsuka Y. Teng K. W.. (2017). Super-resolution imaging of synaptic and extra-synaptic AMPA receptors with different-sized fluorescent probes. elife 6. doi: 10.7554/elife.27744 PMC5779237 28749340

[ref26] Lee M.-C. Yasuda R. Ehlers M. D. (2010). Metaplasticity at single glutamatergic synapses. Neuron 66, 859–870. doi: 10.1016/j.neuron.2010.05.015, PMID: 20620872 PMC2911980

[ref27] Linkert M. Rueden C. T. Allan C. Burel J.-M. Moore W. Patterson A.. (2010). Metadata matters: access to image data in the real world. J. Cell Biol. 189, 777–782. doi: 10.1083/jcb.201004104, PMID: 20513764 PMC2878938

[ref28] MacGillavry H. D. Song Y. Raghavachari S. Blanpied T. A. (2013). Nanoscale scaffolding domains within the postsynaptic density concentrate synaptic AMPA receptors. Neuron 78, 615–622. doi: 10.1016/j.neuron.2013.03.009, PMID: 23719161 PMC3668352

[ref29] Nair D. Hosy E. Petersen J. D. Constals A. Giannone G. Choquet D.. (2013). Super-resolution imaging reveals that AMPA receptors inside synapses are dynamically organized in SSDs regulated by PSD95. J. Neurosci. 33, 13204–13224. doi: 10.1523/jneurosci.2381-12.2013, PMID: 23926273 PMC6619720

[ref30] Ovesný M. Křížek P. Borkovec J. Švindrych Z. Hagen G. M. (2014). Thunderstorm: a comprehensive imagej plug-in for palm and storm data analysis and super-resolution imaging. Bioinformatics 30, 2389–2390. doi: 10.1093/bioinformatics/btu202, PMID: 24771516 PMC4207427

[ref31] Pennacchietti F. Vascon S. Nieus T. Rosillo C. Das S. Tyagarajan S. K.. (2017). Nanoscale molecular reorganization of the inhibitory postsynaptic density is a determinant of GABAergic synaptic potentiation. J. Neurosci. 37, 1747–1756. doi: 10.1523/jneurosci.0514-16.2016, PMID: 28073939 PMC6589977

[ref32] Ramsey A. M. Tang A. H. LeGates T. A. Gou X. Z. Carbone B. E. Thompson S. M.. (2021). Subsynaptic positioning of ampars by LRRTM2 controls synaptic strength. Science Advances 7. doi: 10.1126/sciadv.abf3126 PMC8378824 34417170

[ref33] Rizk A. Paul G. Incardona P. Bugarski M. Mansouri M. Niemann A.. (2014). Segmentation and quantification of subcellular structures in fluorescence microscopy images using Squassh. Nat. Protoc. 9, 586–596. doi: 10.1038/nprot.2014.037, PMID: 24525752

[ref34] Rust M. J. Bates M. Zhuang X. (2006). Sub-diffraction-limit imaging by stochastic optical reconstruction microscopy (STORM). Nat. Methods 3, 793–796. doi: 10.1038/nmeth929, PMID: 16896339 PMC2700296

[ref35] Sando R. Bushong E. Zhu Y. Huang M. Considine C. Phan S.. (2017). Assembly of excitatory synapses in the absence of glutamatergic neurotransmission. Neuron 94, 312–321.e3. doi: 10.1016/j.neuron.2017.03.047, PMID: 28426966 PMC5521186

[ref36] Schiavo G. G. Benfenati F. Poulain B. Rossetto O. de Laureto P. P. DasGupta B. R.. (1992). Tetanus and botulinum-B neurotoxins block neurotransmitter release by proteolytic cleavage of Synaptobrevin. Nature 359, 832–835. doi: 10.1038/359832a0, PMID: 1331807

[ref37] Schiavo G. Matteoli M. Montecucco C. (2000). Neurotoxins affecting neuroexocytosis. Physiol. Rev. 80, 717–766. doi: 10.1152/physrev.2000.80.2.717, PMID: 10747206

[ref38] Sigler A. Oh W. C. Imig C. Altas B. Kawabe H. Cooper B. H.. (2017). Formation and maintenance of functional spines in the absence of presynaptic glutamate release. Neuron 94, 304–311.e4. doi: 10.1016/j.neuron.2017.03.029, PMID: 28426965 PMC5418202

[ref39] Sinnen B. L. Bowen A. B. Forte J. S. Hiester B. G. Crosby K. C. Gibson E. S.. (2017). Optogenetic control of synaptic composition and function. Neuron 93, 646–660.e5. doi: 10.1016/j.neuron.2016.12.037, PMID: 28132827 PMC5300939

[ref40] Sun S.-Y. Li X.-W. Cao R. Zhao Y. Sheng N. Tang A.-H. (2022). Correlative assembly of subsynaptic nanoscale organizations during development. Front. Syn. Neurosci. 14. doi: 10.3389/fnsyn.2022.748184, PMID: 35685244 PMC9171000

[ref41] Tang A.-H. Chen H. Li T. P. Metzbower S. R. MacGillavry H. D. Blanpied T. A. (2016). A trans-synaptic nanocolumn aligns neurotransmitter release to receptors. Nature 536, 210–214. doi: 10.1038/nature19058, PMID: 27462810 PMC5002394

[ref42] Varoqueaux F. Sigler A. Rhee J.-S. Brose N. Enk C. Reim K.. (2002). Total arrest of spontaneous and evoked synaptic transmission but normal synaptogenesis in the absence of MUNC13-mediated vesicle priming. Proc. Natl. Acad. Sci. 99, 9037–9042. doi: 10.1073/pnas.122623799, PMID: 12070347 PMC124419

[ref43] Verderio C. Coco S. Bacci A. Rossetto O. De Camilli P. Montecucco C.. (1999). Tetanus toxin blocks the exocytosis of synaptic vesicles clustered at synapses but not of synaptic vesicles in isolated axons. J. Neurosci. 19, 6723–6732. doi: 10.1523/jneurosci.19-16-06723.1999, PMID: 10436029 PMC6782867

[ref44] Verhage M. Maia A. S. Plomp J. J. Brussaard A. B. Heeroma J. H. Vermeer H.. (2000). Synaptic assembly of the brain in the absence of neurotransmitter secretion. Science 287, 864–869. doi: 10.1126/science.287.5454.864, PMID: 10657302

[ref45] Wang Y. Schnitzbauer J. Hu Z. Li X. Cheng Y. Huang Z.-L.. (2014). Localization events-based sample drift correction for localization microscopy with redundant cross-correlation algorithm. Opt. Express 22:15982. doi: 10.1364/oe.22.015982 24977854 PMC4162368

[ref46] Yang X. Le Corronc H. Legendre P. Triller A. Specht C. G. (2021). Differential regulation of glycinergic and GABAergic nanocolumns at mixed inhibitory synapses. EMBO Rep. 22:e52154. doi: 10.15252/embr.202052154, PMID: 34047007 PMC8256292

[ref47] Zeng M. Díaz-Alonso J. Ye F. Chen X. Xu J. Ji Z.. (2019). Phase separation-mediated TARP/MAGUK complex condensation and AMPA receptor synaptic transmission. Neuron 104, 529–543.e6. doi: 10.1016/j.neuron.2019.08.001, PMID: 31492534 PMC6842113

[ref48] Zhu Y. Uytiepo M. Bushong E. Haberl M. Beutter E. Scheiwe F.. (2021). Nanoscale 3D em reconstructions reveal intrinsic mechanisms of structural diversity of chemical synapses. Cell Rep. 35:108953. doi: 10.1016/j.celrep.2021.108953, PMID: 33826888 PMC8354523

