# Erratum: AMPA and GABAA receptor nanodomains assemble in the absence of synaptic neurotransmitter release

**DOI:** 10.3389/fnmol.2023.1281653

**Published:** 2023-09-01

**Authors:** Frontiers Production Office

**Affiliations:** Frontiers Media SA, Lausanne, Switzerland

**Keywords:** Synapse, neurotransmitter release, postsynaptic neurotransmitter receptors, AMPA receptor, GABA receptor, excitatory, inhibitory, nanodomain

Due to a production error, there was a mistake in the legends for Figures 2–4 as published. The requested changes were missed during typesetting. The correct legends appear below.

**Figure 2:**
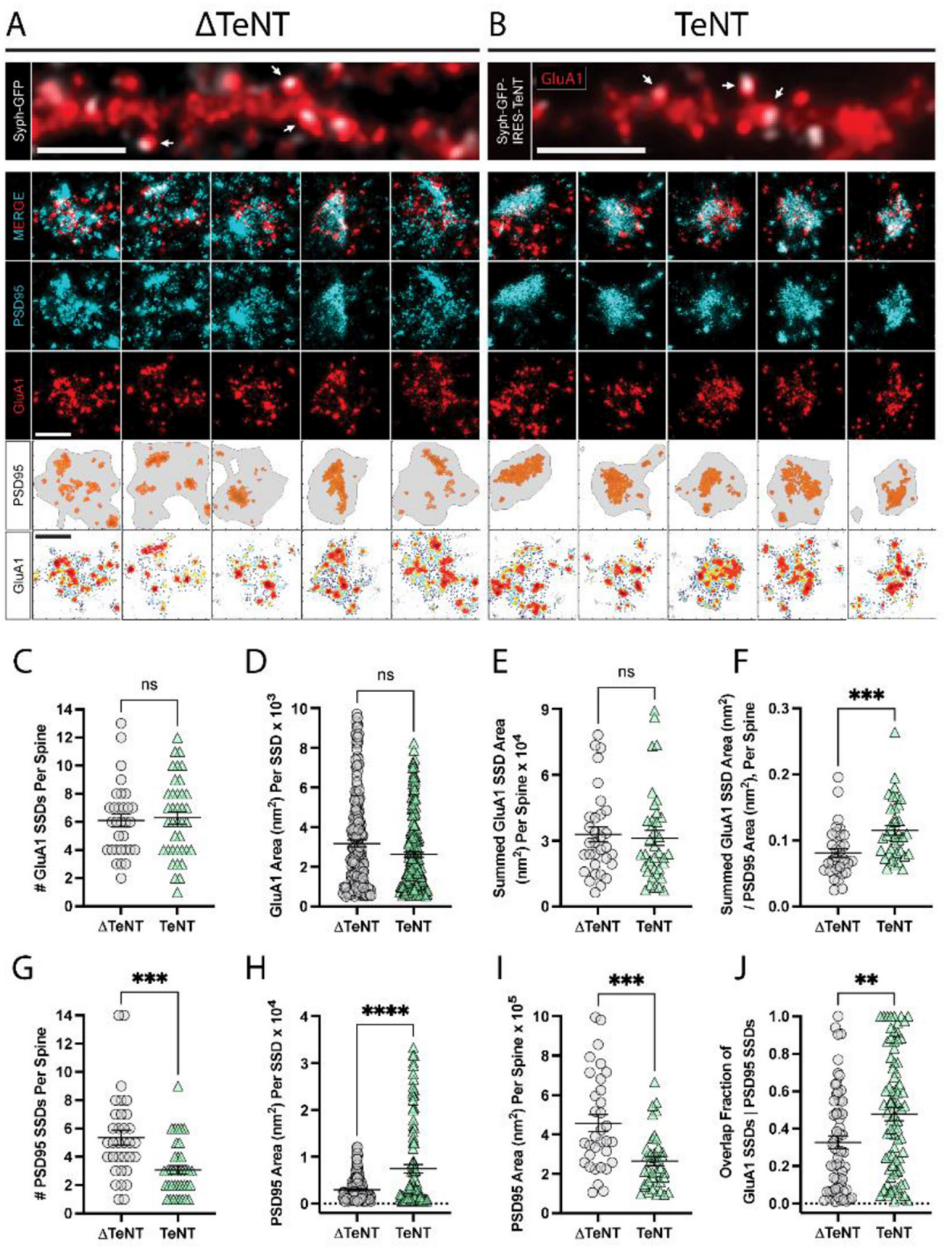
Formation of excitatory receptor and scaffold SSDs in the absence of neurotransmission. **(A, B)** Representative epifluorescence images of dendritic regions for cultures infected with control virus (ΔTeNT, **A**), or TeNT virus (TeNT, **B**) and stained for surface GluA1 (red) are shown (top panels) with syph-GFP, or syph-GFP-IRES-TeNT shown in greyscale. In both cases note the apposition of syph-GFP signal and GluA1-positive (red) dendritic spines (white arrows). Scale bar = 5 μm in each image. Representative dSTORM images of synapses contacted by ΔTeNT **(A)**, or TeNT **(B)** terminals are shown below in rows 2–4. Row 5 shows the PSD boundary for each synapse, defined by a minimum PSD95 localization density (gray borders), with PSD95 localizations in defined SSDs rendered in orange. Row 6 shows GluA1 localization density maps for each synapse with warmer colors representing higher GluA1 localization density. Scale bars in rows 4 and 6 both = 500 nm. **(C)** There was no significant difference in the number of GluA1 SSDs per spine at TeNT-silenced synapses compared to controls [spines, *n* = 31 ΔTeNT/38 TeNT; ns, not significant; *p* = 0.7690 (*T*-Test)]. **(D)** The area of individual GluA1 SSDs was not significantly different at TeNT-silenced synapses compared to controls [SSDs, *n* = 194 ΔTeNT/200 TeNT; ns, not significant; *p* = 0.0531 (Mann–Whitney)]. **(E)** The summed GluA1 SSD area, per spine was not significantly different at TeNT-silenced synapses compared to controls [spines, *n* = 32 ΔTeNT/38 TeNT; ns, not significant; *p* = 0.5777 (Mann–Whitney)]. **(F)** The ratio between the summed GluA1 SSD area to its total PSD95 area was significantly increased at TeNT-contacted dendritic spines [spines, *n* = 33 ΔTeNT/37 TeNT; ^***^p < 0.001; *p* = 0.0007 (Mann–Whitney)]. **(G)** The number of PSD95 SSDs per spine was significantly decreased at TeNT-contacted spines compared to controls [spines, *n* = 33 ΔTeNT/38 TeNT; ^***^p < 0.001; *p* = 0.0002 (Mann–Whitney)]. **(H)** The area of individual PSD95 SSDs was significantly increased at TeNT-silenced spines compared to controls [SSDs, *n* = 141 ΔTeNT/103 TeNT; ^****^p < 0.0001 (Welch's *T*-Test)]. **(I)** The total PSD95 area per spine was significantly reduced at TeNT-contacted spines compared to controls [spines, *n* = 33 ΔTeNT/38 TeNT; ^***^p = 0.0001 (Mann–Whitney)]. **(J)** The degree of GluA1 SSD/PSD95 SSD overlap was significantly increased at TeNT-contacted spines compared to controls [SSDs, *n* = 61 ΔTeNT/81 TeNT; ^**^p < 0.01; *p* = 0.0052 (Mann–Whitney)].

**Figure 3:**
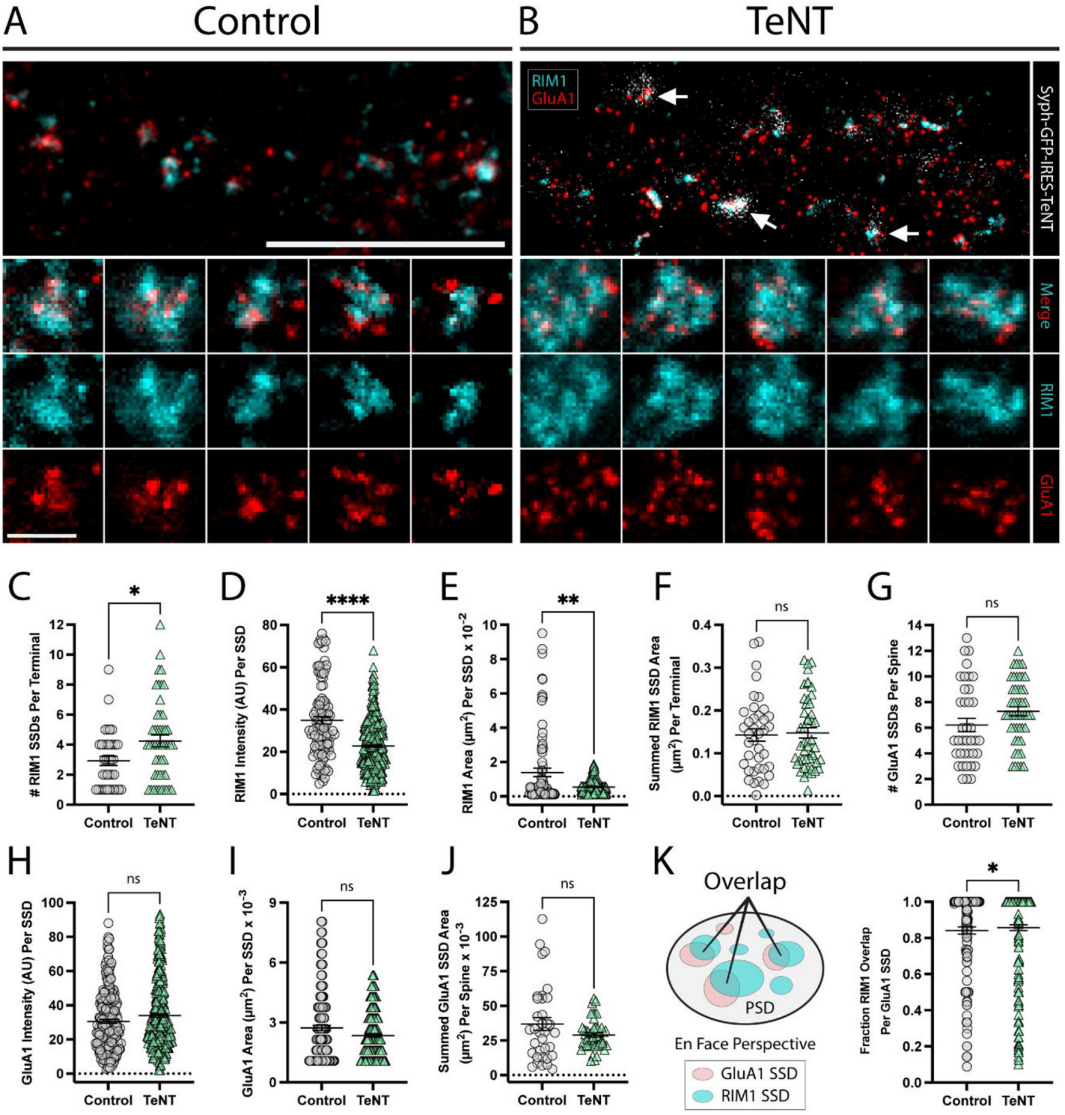
Trans-synaptic SSD alignment is intact at chronically silenced excitatory synapses. **(A, B)** Representative STED images of synapses labeled with RIM1 (cyan) and GluA1 (red) for (control, **A**) and (TeNT, **B**) conditions. Note the apposition of syph-GFP signal and GluA1-positive (red) dendritic spines (white arrows). Scale bar = 5 μm. Columns 1–5 in **(A, B)** display representative STED images of synapses for control **(A)**, and TeNT **(B)** conditions; merged RIM1 (cyan), and GluA1 (red) (row 2); RIM1 (cyan) (row 3); GluA1 (red) (row 4). Scale bar = 500 nm. **(C)** The number of RIM1 SSDs per synaptic terminal was significantly increased at TeNT-expressing terminals compared to controls [terminals, *n* = 38 control/49 TeNT; ^*^p < 0.05 (Mann–Whitney)]. **(D)** RIM1 fluorescence intensity quantified at individual SSDs was decreased at TeNT-silenced terminals compared to controls [SSDs, *n* = 104 control/346 TeNT; ^****^p < 0.0001 (Mann–Whitney)]. **(E)** The area of individual RIM1 SSDs was decreased at TeNT-silenced terminals compared to controls [SSDs, *n* = 81 control/281 TeNT; ^**^p < 0.01 (Welch's *T*-Test)]. **(F)** The summed RIM1 SSD area per terminal was not significantly different at TeNT-silenced terminals compared to controls [terminals, *n* = 38 control/48 TeNT; ns, not significant; *p* = 0.7050 (Mann–Whitney)]. **(G)** The number of GluA1 SSDs per spine was not significantly different at TeNT-silenced spines compared to controls [spines, *n* = 38 control/49 TeNT; ns, not significant; *p* = 0.0539 (Mann–Whitney)]. **(H)** The fluorescence intensity of individual GluA1 SSDs was not significantly different at TeNT-silenced spines compared to controls [SSDs, *n* = 218 control/372 TeNT; ns, not significant; *p* = 0.0959 (Mann–Whitney)]. **(I)** The mean area of individual GluA1 SSDs was not significantly different at TeNT-silenced spines compared to controls [SSDs, *n* = 187 control/339 TeNT; ns, not significant; *p* = 0.2098 (Mann–Whitney)]. **(J)** The summed GluA1 SSD area per spine was not significantly different at TeNT-silenced spines compared to controls [spines, *n* = 35 control/49 TeNT; ns, not significant; *p* = 0.1120 (Welch's *T*-Test)]. **(K)** Schematic showing the fraction of GluA1/RIM1 SSD overlap (left). GluA1 SSD overlap with RIM1 SSDs was slightly elevated at TeNT-associated synapses (i.e., on average, a larger area of each GluA1 SSD at TeNT-associated synapses was overlapped by closely-associated RIM1 SSDs) [SSDs, *n* = 155 Control/268 TeNT; ^*^p < 0.05; *p* = 0.0372 (Mann–Whitney)].

**Figure 4:**
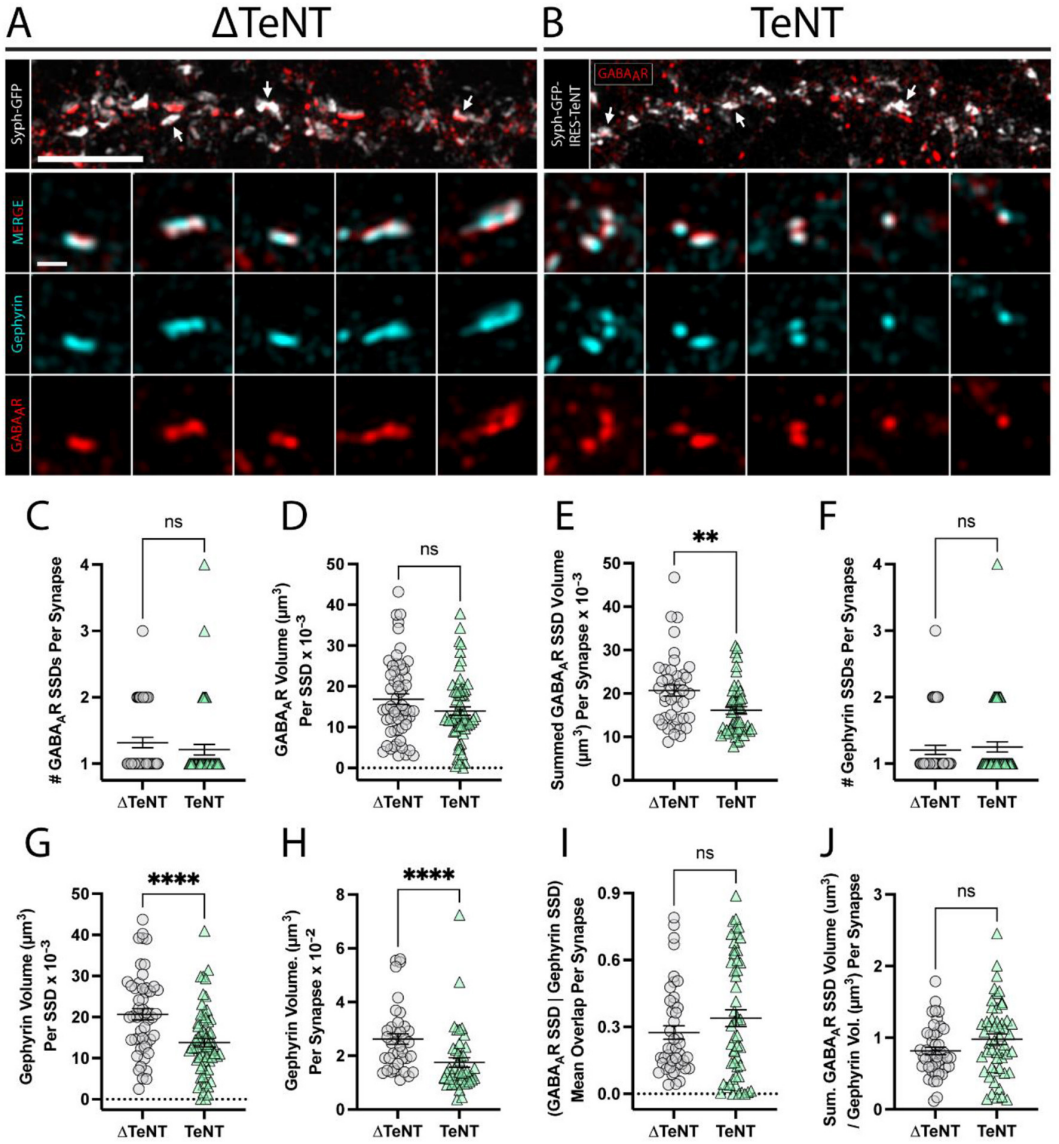
Formation of inhibitory receptor and scaffold SSDs in the absence of neurotransmission. **(A, B)** Representative SIM images of dendritic regions from (ΔTeNT, **A**) or (TeNT, **B**), cultures displaying syph-GFP, or syph-GFP-IRES-TeNT (greyscale), and GABAAR (red). Note the apposition of syph-GFP signal and GABAAR-positive (red) synapses (white arrows). Scale bar = 5 μm. Columns 1–5 display representative synapses for ΔTeNT **(A)**, and TeNT **(B)** conditions; merged gephyrin (cyan), and GABAAR (red) (row 2); gephyrin (cyan) (row 3); and GABAAR (red) (row 4). Scale bar = 500 nm. **(C)** The number of GABAAR SSDs per synapse was not significantly different at TeNT-silenced synapses compared to controls [synapses, *n* = 44 ΔTeNT/52 TeNT; ns, not significant; *p* = 0.1340 (Mann–Whitney)]. **(D)** The volume of individual GABAAR SSDs was not significantly different at TeNT-silenced synapses compared to controls [SSDs, *n* = 57 ΔTeNT/63 TeNT; ns, not significant; *p* = 0.0739 (Mann–Whitney)]. **(E)** The summed synaptic volume of GABAAR SSDs per synapse was significantly decreased at TeNT-silenced spines compared to controls [synapses, *n* = 41 ΔTeNT/50 TeNT; ^**^p < 0.01; *p* = 0.0020 (Mann–Whitney)]. **(F)** The number of gephyrin SSDs per synapse was not significantly different at TeNT-silenced synapses compared to controls [synapses, *n* = 44 ΔTeNT/52 TeNT; ns, not significant; *p* = 0.7200 (Mann–Whitney)]. **(G)** The volume of individual gephyrin SSDs was significantly reduced at TeNT-silenced synapses compared to controls [SSDs, *n* = 52 ΔTeNT/64 TeNT; ^****^p < 0.0001 (*T*-Test)]. **(H)** The total gephyrin volume per synapse was significantly decreased at TeNT-silenced synapses compared to controls [synapses, *n* = 38 ΔTeNT/44 TeNT; ^****^p < 0.0001 (Mann–Whitney)]. **(I)** There was no significant difference in the mean overlap between GABAAR SSDs and gephyrin SSD signals at TeNT-silenced synapses compared to controls [SSDs, *n* = 43 ΔTeNT/52 TeNT; ns, not significant; *p* = 0.5039 (Mann–Whitney)]. **(J)** The ratio between the summed synaptic volume of GABAAR SSDs, and the total gephyrin volume, per synapse was not significantly different at TeNT-silenced synapses compared to controls [synapses, *n* = 44 ΔTeNT/51 TeNT; ns, not significant; *p* = 0.0795 (*T*-Test)].

The publisher apologizes for this mistake. The original article has been updated.

